# A Therapeutic Perspective of HDAC8 in Different Diseases: An Overview of Selective Inhibitors

**DOI:** 10.3390/ijms231710014

**Published:** 2022-09-02

**Authors:** Anna Fontana, Ilaria Cursaro, Gabriele Carullo, Sandra Gemma, Stefania Butini, Giuseppe Campiani

**Affiliations:** 1 Department of Biotechnology, Chemistry and Pharmacy, University of Siena, Via Aldo Moro 2, 53100 Siena, Italy; 2 Department of Life Sciences, University of Siena, Via Aldo Moro 2, 53100 Siena, Italy

**Keywords:** histone deacetylase, HDAC8, HDAC8 inhibitor, fibrosis, cancer, polypharmacology

## Abstract

Histone deacetylases (HDACs) are epigenetic enzymes which participate in transcriptional repression and chromatin condensation mechanisms by removing the acetyl moiety from acetylated ε-amino group of histone lysines and other non-histone proteins. In recent years, HDAC8, a class I HDAC, has emerged as a promising target for different disorders, including X-linked intellectual disability, fibrotic diseases, cancer, and various neuropathological conditions. Selective HDAC8 targeting is required to limit side effects deriving from the treatment with *pan*-HDAC inhibitors (HDACis); thus, many endeavours have focused on the development of selective HDAC8is. In addition, polypharmacological approaches have been explored to achieve a synergistic action on multi-factorial diseases or to enhance the drug efficacy. In this frame, proteolysis-targeting chimeras (PROTACs) might be regarded as a dual-targeting approach for attaining HDAC8 proteasomal degradation. This review highlights the most relevant and recent advances relative to HDAC8 validation in various diseases, providing a snapshot of the current selective HDAC8is, with a focus on polyfunctional modulators.

## 1. Introduction

Histone deacetylases (HDACs), also known as lysine deacetylases (KDACs), belong to the class of zinc (Zn^2+^)-dependent or nicotinamide adenine dinucleotide (NAD^+^)-dependent proteolytic enzymes. HDACs participate in transcriptional repression and chromatin condensation mechanisms by removing the acetyl moiety from the acetylated ε-amino group of histone lysines and other non-histone proteins [1]. These enzymes play a pivotal role in the modulation of several cellular pathways such as cell proliferation, apoptosis, neurogenesis and epigenetic regulations [2]. In some cases, HDACs are involved in the occurrence and progression of numerous pathophysiological conditions as well as diseases such as neurological disorders, fibrosis, cancer, metabolic dysfunctions and parasitic infections [2,3,4,5,6,7].

## 2. HDAC8 Is a Class I HDAC Enzyme

HDAC enzymes are categorized into two groups: the Zn^2+^-dependent enzymes, namely class I (HDAC1, 2, 3 and 8), class II (HDAC4, 5, 6, 7, 9 and 10), class IV (HDAC11), and the NAD^+^-dependent enzymes (class III, also known as sirtuins) [8]. Exclusive features of the HDAC8 isoform are that its gene lies in the X chromosome and that it holds key structural differences as compared to other class I HDACs. HDAC8 is smaller than HDACs 1–3 and has an independent behaviour because it lacks the C-terminal protein–protein interaction domains able to promote the formation of multiprotein complexes [9]. Over the years, it has been demonstrated that HDAC8 has a lower turnover number/concentration of substrate which permits the enzyme to have a lower specificity constant (*k*_cat_/*K*_m_) value for the hydrolysis of acetyl lysine-containing peptides compared to HDAC1 or HDAC6, although HDAC8 presents a significant catalytic activity against tetrameric histone H3/H4 proteins [10]. Other HDAC8 substrates have been identified such as p53, oestrogen-related receptor α (ERRα), AT-rich interactive domain-containing protein 1A (ARID1a), and the structural maintenance of chromosomes 3 protein (SMC3). This latter protein is the only substrate exclusively and directly deacetylated by HDAC8. Nevertheless, HDAC8 may also deacetylate other candidates by recruiting these proteins in complexes, as in the case of inv(16) fusion protein [11]. It has been shown that HDAC8 hydrolyses acyl lysine residues in peptides having acyl chains of 2−16 carbons, with higher values of *k*_cat_/*K*_m_ for the longer octanoyl, dodecanoyl-, and myristoyl-acyl chains. These long chains are able to accommodate in the binding cleft extending beyond the active site Zn^2+^ cation to occupy the hydrophobic internal cavity lateral to the substrate channel. Fatty acid deacylation (removal of acyl groups with 2–16 carbons from octanoyl, dodecanoyl, and myristoyl lysine) might be a more physiologically relevant yet broad HDAC8 activity with respect to histone deacetylation, explaining how there is a small number of in vivo relevant HDAC8 substrates [12].

**Figure 1:**
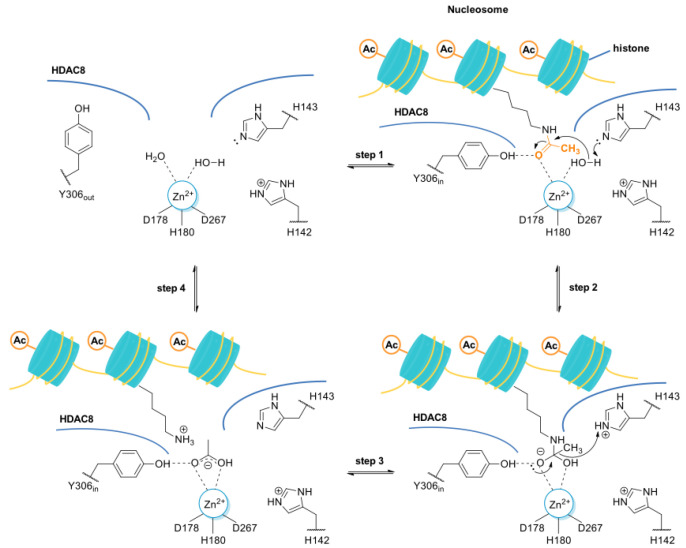
Proposed deacetylation mechanism of *N*-acetyl lysine residue of histone protein by the HDAC8 enzyme. The residue H143, acting as a base, deprotonates a Zn^2+^-bound water molecule, which, in turn, performs the nucleophilic attack at the Zn^2+^-coordinated carbonyl group of the *N*-acetyl-lysine (step 1 and 2). The tetrahedral intermediate and its transition states are stabilized by the coordination bond with the Zn^2+^ along with the hydrogen bond interactions with Y306, H143, and H142. H143, acting as acid catalyst, assists the rearrangement of the tetrahedral intermediate to give the positively charged lysine (step 3 and 4). It was hypothesized that the side chain of Y306 may undergo conformational changes, adopting the “out” conformation in absence of the substrate, while the “in” conformation is required for the substrate binding and catalysis.

The first HDAC8 crystal structure was described by Somoza et al. in 2004 [13]. In particular, the carbonyl group of the acetyl-*L*-lysine coordinates with the Zn^2+^ ion and forms a hydrogen bond with Y306 of human HDAC8 (*h*HDAC8) [14]. Furthermore, the tandem histidine pair formed by residues H142 and H143 is crucial to stabilize the transition state. H142 serves as the general base catalyst, whereas H143 serves as both the general acid and the general base catalyst (Figure 1) [15,16].

**Figure 2:**
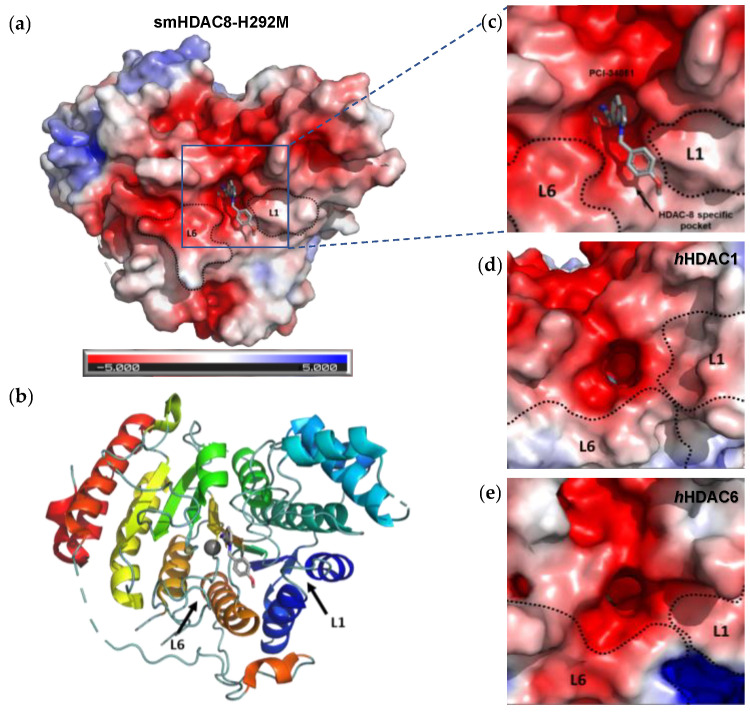
Representation of *sm*HDAC8-H292M in complex with **1** (PDB code: 6HSF), shown as (**a**) electrostatic potential surfaces, and (**b**) ribbon and sticks structure. Delineation of the L1 and L6 loops forming the walls of the catalytic pocket of HDAC8, HDAC1, and HDAC6: close-up representation of the electrostatic potential surfaces of (**c**) crystal structure of mutant *sm*HDAC8-H292M in complex with compound **1** (PDB code: 6HSF), (**d**) crystal structure of *h*HDAC1 (PDB code: 4BXK), (**e**) crystal structure of *h*HDAC6 catalytic domain 2 (CD2) (PDB code: 5EDU). PCI-34051 (**1**) is depicted in light grey. Co-crystal structures were downloaded from the Protein Data Bank and visualized by Pymol (PyMOL^TM^ 2.5.1).

It was also observed that there is a conserved glycine-rich loop (G302GGGY) that is crucial for the maintenance of the Y306 residue. In addition, the glycine residues, especially G304 and G305, provide flexibility to the G302GGGY loop [17]. The active site tunnel of HDAC8 is constituted of residues such as G151, F152, H180, F208, M274 and Y306. These amino acid residues are generally hydrophobic and conserved in the class I HDACs. These amino acids help during the binding of an inhibitor containing bulky hydrophobic linker to the HDAC8 enzyme. Y306 cooperates with some aminoacidic residues of L6 and L1 loops, creating a distinctive sub-pocket, whose structural features are widely exploited in the drug design of selective HDAC8 inhibitors (HDAC8is). Compounds that assume a low energy L-shaped conformation make additional contacts with the HDAC8 catalytic domain, in comparison with other class I HDACs. In this context, PCI-34051 (**1**, Figure 2), a selective and potent HDAC8i, was extensively studied for its ability to discriminate among the other isoforms (HDAC1 IC_50_ = 4 μM, HDAC6 IC_50_ = 2.9 μM, and HDAC8 IC_50_ = 0.01 μM), allowing for attainment of crucial information concerning the binding mode [18,19].

However, selective HDAC8 inhibition over HDAC6 proved to be more challenging, due to the structural similarities in the active site [20,21]. In contrast with HDAC8, L6 and L1 loops in HDAC6 isoform are able to form a larger and shallower groove that preferentially accommodates Y-shaped inhibitors endowed with extended and bulky cap groups (Figure 2) [22,23]. Briefly, the principal differences that define HDAC8 as a unique isoform are the following:

- HDAC8 is an X-linked protein which acts independently, e.g., without forming any co-complexes for the activity;
- The L1 loop of HDAC8 is closest to the enzyme active site and undergoes conformational changes, differently depending on the substrate (Figure 2);
- L1 and L6 form a specific pocket which requires an “L” shape conformation for selective binding (Figure 2);
- HDAC8 presents a nuclear localization sequence between the catalytic domain of the enzyme and the serine binding motif found at the end of the catalytic domain [24].

### 2.1. HDAC8 Substrates

The typical HDAC8 histone substrates include full-length H2A/H2B, H3, and H4 histones acetylated at nonspecific lysine residues. Peptide sequences corresponding to the H4 histone tail with an acetylated lysine at position sixteen [K(ac)16] were investigated as suitable in vitro substrates [25]. HDAC8 also catalyses in vitro deacetylation of the K(ac)20 site on the H4 histone tail. However, HDAC8-catalysed deacetylation of the K(ac)20 peptide is much slower than deacetylation of K(ac)16 peptides [26]. Global histone acetylation levels could mask the specific deacetylation pattern of H3 by HDAC8, suggesting that non-histone targets are necessary to ascertain the exact role of HDAC8 in pathophysiological conditions. Non-histone targets are proteins whose activity could be used as a measure of HDAC activity in cells. The most important non-histone substrates identified thus far are SMC3, p53, ERRα, inv(16) and cAMP response element-binding protein (CREB). In most of these proteins, HDAC8 exhibits selectivity for certain deacetylation sites, such as arginine-Kac129 (RKac) in ERRα, RSKacFE in inv(16) fusion protein, and RHKK in p53. In general, the *N*-terminal arginine at position −1 to the K(ac) and an aromatic ring (e.g., phenylalanine) at the C-terminal position +1 constitute the most effective deacetylation sites [27].

#### 2.1.1. SMC3

SMC3 is one of the most important HDAC8 substrates, with clear concentration-dependent hyperacetylation effects. Generally, SMC3 forms a clutch to hold the sister chromatids together during cell cycle progression [28]. Its primary deacetylation sites are conserved K105 and K106 (human numbering). The deacetylation of SMC3 is important to segregate the sister chromatids in early mitosis and to reload the SMC3 pool for a new cell cycle. Loss of HDAC8 deacetylation activity leads to accumulation of acetylated SMC3 (Ac-SMC3) with decreased affinity towards chromatids, ultimately affecting gene transcription [10].

#### 2.1.2. p53

The tumour suppressor p53 requires HDAC8 for its expression. Depletion of HDAC8 decreases homeobox A5 (HoxA5)-dependent expression of wild-type (WT) and mutant p53 mice. The ectopic manifestation of HDAC8 increases p53 transcription while HDAC8 inactivation may be effective for p53 mutant tumour cells. Silencing or inhibition of HDAC8 mainly affects the proliferation of those cells harbouring a p53 mutation. Thus, it is reasonable to hypothesize that HDAC8is could be useful as adjuvants for the treatment of tumours carrying a p53 mutation [10,29].

#### 2.1.3. ERRα

Acetylation state of ERRα can be detected at four lysine residues, where post-translational modifications, as deacetylation, inhibit the DNA binding and the ERRα transcriptional activity [30]. Furthermore, incubation of purified acetylated-ERR*α* with HDAC8 enhances the affinity of ERR*α* for DNA, which is consistent with HDAC8-catalysed deacetylation of ERRα. The acetylation site (K129(ac)) in ERRα has R121, facilitating HDAC8-mediated catalysis. [31].

#### 2.1.4. inv(16)

HDAC8 co-localizes and immunoprecipitates with smooth muscle myosin heavy chain, suggesting that HDAC8 may interact with this domain within the inv(16) fusion protein [11]. When HDAC8 is overexpressed, it associates with inv(16). As a consequence, HDAC8 contributes to the transcriptional repression mediated by this chromosomal translocation fusion protein. Other HDACs do not immunoprecipitate with inv(16), suggesting that HDAC8 may be the main HDAC isoform interacting with inv(16) in vivo [32]. 

#### 2.1.5. CREB

HDAC8 and CREB are overexpressed in HEK293 cells, and they can be immunoprecipitated together, demonstrating that the functions of these proteins are associated. When HDAC8 is overexpressed in cells, phosphorylation of CREB decreases, which in turn inhibits CREB transcriptional activation. As HDAC overexpression can affect several targets within the cell, this inhibition may indirectly affect CREB phosphorylation. Nevertheless, CREB is not a selective partner for HDAC8 isoenzyme [33].

α-tubulin (a well-known HDAC6 acetylation substrate) was also reported as a marker of HDAC8 inhibition, limiting the possibility to discriminate between HDAC6 and HDAC8 selective inhibition [21,34,35]. Different studies demonstrated that the measurement of H3 acetylation levels should be the method of choice for more reliably assessing selective HDAC8 inhibition with respect to HDAC6 inhibition [36,37].

To date, amongst the non-histone proteins, SMC3 has been acknowledged as the preferential HDAC8 acetylation substrate, while H3 represents the main histone target for HDAC8. Therefore, selective target engagement is increasingly studied by evaluating their level of acetylation, both in vitro and in vivo experiments.

## 3. Involvement of HDAC8 in Different Diseases

Altered HDAC8 expression has been related to different pathological conditions, generally depending on substrates (histone or non-histone) and their abnormal level of deacetylation. HDAC8 engagement can occur as a loss of enzymatic activity that requires the treatment with activators [38,39]. Nevertheless, in most disorders, HDAC8 has an unregulated or excessive activity that can be counteracted by inhibitors. In the next sections, we analyse the role of HDAC8 in specific diseases and, when ascertained, its mechanism of action. 

### 3.1. X-Linked Disorders

Mutations on specific genes harboured in the X-chromosome are responsible for neurodevelopmental disorders, such as Cornelia de Lange syndrome (CdLS) and Duchenne muscular dystrophy (DMD). Several studies have enlightened that HDAC8 is enrolled in both diseases in different ways, confirming the versatile nature of this target [27]. Of note, the most innovative therapeutic approaches, such as gene therapy, are not sufficient to restore the most common mutations in X-linked disorders. More in detail, the systemic nature of these pathologies requires body-wide gene transfer of non-pathogenic viral vectors, whose high dosage could cause adverse immune response [40,41]. Thus, different strategies should be pursued to achieve an improvement in the life quality and life expectancy of affected patients.

#### 3.1.1. Cornelia de Lange Syndrome (CdLS)

CdLS is a rare genetic disorder that leads to prenatal and postnatal growth retardation, congenital malformations, distinctive facial feature, intellectual disability [42]. In general, CdLS is caused by different mutations in genes encoding proteins that regulate the cohesin complex (Nipped-B-like protein (NIPBL) and HDAC8) or its core components, (double-strand-break repair protein 21 (RAD21), SMC1A and SMC3). Mutations on the HDAC8 gene account for almost 5% of cases that display a similar phenotype, classified as CdLS 5 (OMIM#300882) [43,44]. Patients showed a partial or complete loss of deacetylase activity, depending on the mutation localization in the gene encoding HDAC8 [42]. The resulting accumulation of Ac-SMC3, a bona fide HDAC8 substrate, limits the segregation of the sister chromatids and the recycling of cohesin for another cell cycle. These events lead to a cohesin-mediated transcriptional dysregulation. CdLS is mainly treated by limiting the disease progression and related symptoms [45]. Medicinal chemistry efforts have been focused on the identification of HDAC8 activators to restore the residual deacetylase activity [39]. 

**Figure 3:**
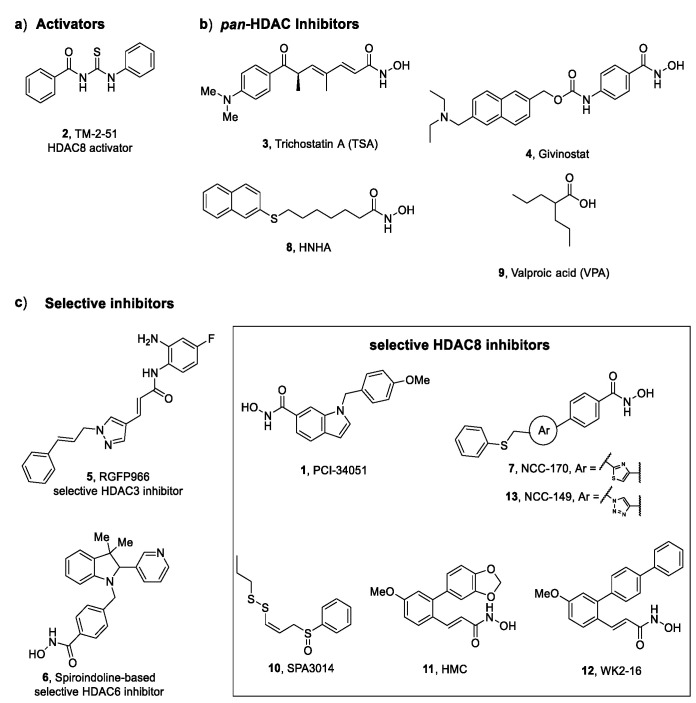
HDAC activators (**2**) and inhibitors (**1, 3**-**13**) explored in key pathologies: (**a**) thiourea-based activator of HDAC8, (**b**) *pan*-HDAC inhibitors, (**c**) selective HDAC inhibitors.

A series of *N*-acetylthioureas proved effective in increasing HDAC8 activity in inhibitory enzymatic assay; thus, further studies were performed for the most potent activator, TM-2–51 (**2**, Figure 3). Singh et al. demonstrated that this compound is able to increase the catalytic turnover rate of the enzyme and to enhance HDAC8 catalysed reactions by 16-fold at 10 μM concentration, by decreasing the *K*_m_ value of the substrate [46]. In addition, computational studies were performed to elucidate the pharmacodynamic profile of **2** and its mechanism of activation. The results suggested that the agonist binds to the HDAC8 enzyme in the proximity of the active site, hypothesizing the presence of an allosteric site [46]. Molecular dynamic simulations proposed a “sandwich-like” binding capable of stabilizing the enzyme complex only in the presence of “loosely bound” substrates [39]. The evaluation of the activity of **2** on certain CdLS HDAC8 mutants highlighted the ability of the activator to rescue the catalytic activity to wild-type levels [47]. This latter finding supports the potential use of HDAC8 activators as therapeutic tools to attenuate the progression of the neurodevelopmental impairments and related deformities in CdLS patients diagnosed with HDAC8 mutation.

#### 3.1.2. Duchenne Muscular Dystrophy (DMD)

DMD is a severe muscle-wasting disorder whose development is ascribable to mutations in the gene (DMD) encoding dystrophin that prevent the production of this protein in the striated and cardiac muscles. Despite the significant advances in insights of pathogenesis, the current therapeutic treatment is mostly aimed at relieving the symptoms. The current therapy consists of administration of corticosteroids, orthopaedic surgery, and eventually assisted ventilation [48,49]. Different studies have enlightened the ability of *pan*-HDACis to interfere with the pathogenesis of DMD. In particular, the treatment of a DMD mouse model (*mdx* mice) and zebrafish model with Trichostatin A (**3**, TSA, Figure 3) ameliorated the resistance to the process of degeneration and regeneration promoted by contraction and led to the rescue of the fibre damage [50,51]. A preliminary study performed by Consalvi et al. has revealed the beneficial effect of Givinostat (4, Figure 3) in preventing DMD progression in mdx mice. The inhibitor exerts its action on downstream effectors of dystrophin-nitric oxide signalling, leading to a reduction of inflammation and fibrosis in muscle tissue and increasing the cross-sectional area of the myofibers [52]. The study of the potential use of **4** in a long-term treatment of DMD allowed for its selection as a drug candidate for advanced phase clinical trials [53]. Among the epigenetic enzymes, HDAC8 has been proven to be overexpressed in the myotubes from DMD patients and in dystrophin morpholino(*dmd*-MO)-injected zebrafish embryos lacking dystrophin early in development. In the same work, the impact of HDAC8 inhibition on DMD pathogenesis was explored in a DMD zebrafish model by using PCI-34051 (**1**, Figure 3). This highly selective HDAC8 inhibitor was able to rescue the DMD phenotype by restoring skeletal muscle histomorphology and reducing inflammation in a similar manner to Givinostat (**4**). Western Blot analysis was performed to evaluate the acetylation profile of a DMD zebrafish model: the results have demonstrated that the treatment with compound **1** produced an increase in α-tubulin (a marker of microtubules stability). Moreover, the assessment of cytoskeleton architecture in human DMD myoblast confirmed that HDAC8 inhibition has a crucial role in the maintenance of the skeletal muscle histomorphology [54]. Taken together, these results validated HDAC8 as a potential therapeutic target to tackle DMD.

### 3.2. Aberrant Wound Healing

Wound healing is a transient well-orchestrated process that occurs in response to tissue damage. The consequent repair requires a controlled deposition of collagen aimed at promote scarring and tissue regeneration [55]. The dysregulation of the injury-repair mechanisms can result in fibrosis, a chronic pathological condition characterized by excessive accumulation of extracellular matrix (ECM) components, such as collagen and fibronectin, that might impair the normal function of the affected tissue and relative organs [56]. The fibrotic process is associated with diverse diseases that hit different organs such as lungs, kidneys, liver and heart. The causative agent is mostly unknown, even if it is possible to identify asbestos, silica, cigarette smoke, persistent infections, and chronic inflammation as possible factors able to trigger the development of uncontrolled deposition of fibrous connective material. Fibrosis initiates with the differentiation of quiescent fibroblasts, and minimally of circulating fibrocytes, into myofibroblasts. These latter have been identified as the main fibrotic effectors [57,58]. Moreover, some studies highlighted that diverse cell types migrate to the fibrotic tissue where they are activated into myofibroblasts. In particular, epithelial cells enrich the pool of myofibroblasts since they undergo epithelial–mesenchymal transition (EMT), a transdifferentiation induced by transforming growth factor (TGF-β), predominantly through the SMAD3 signalling cascade [59]. These phenotypic conversions are elicited by TGF-β1 (a well-established profibrotic mediator) together with other molecular players, comprising tumour necrosis factor-α (TNF-α), basic fibroblast growth factor (bFGF), and connective tissue growth factor (CTGF). Differently from fibroblasts, myofibroblasts stimulate excessive production of ECM components and upregulate tissue inhibitors of metalloproteinases (TIMPs) which cannot counteract the activity of ECM degradative enzymes [60]. To date, treatment options are limited to cell therapy—consisting in the transplantation of bone marrow-derived mesenchymal stem cells in the lesioned tissue–and the modulation of validated targets by small-molecule drugs [61]. Previous works have investigated the involvement of HDACs in fibrogenesis and how their inhibition might ameliorate the fibrotic tissue where they are overexpressed [60,62]. It was demonstrated that many isoforms can act at specific levels, producing different pro-fibrotic effects, including the increased secretion of pro-fibrotic cytokines, growth factors [63], and the induction of myofibroblast differentiation [64]. HDAC8 has emerged as a valuable target for fibrosis-associated disease, and here, we briefly report the scientific pieces of evidence that support this therapeutic option.

#### 3.2.1. Pulmonary Fibrosis (PF)

PF is a rare, chronic and interstitial lung pathological condition characterized by the progressive replacement of the parenchyma with fibrotic scar tissue [65]. PF leads to an impairment of gas exchange at the alveolar level with consequent dysfunctional breathing which degenerates into respiratory and heart failure. Sometimes, PF is correlated to other lung diseases such as scleroderma, sarcoidosis, and parasitic infection or can be caused by exposure to environmental agents or radiations [66]. Among PF phenotypes, idiopathic pulmonary fibrosis (IPF) is the most disrupting due to its unknown aetiology and poor prognosis. The current therapy is associated with the use of nintedanib (Ofev^®^, Boehringer Ingelheim, Ingelheim, Germany) and pirfenidone (Esbriet^®^, Roche, Basel, Switzerland) that exert their antifibrotic effect by limiting the activation of platelet-derived growth factor receptor and decreasing the production of profibrotic growth factors and cytokines, respectively. Nevertheless, their biological effects are not sufficient to reverse the disorder; thus, new pharmacological approaches should be considered. A rising array of evidence suggests that HDACs modulation could interfere with fibrotic events in IPF [65]. In particular, treatment of preclinical PF models (primary IPF fibroblasts and mouse model of bleomycin-induced lung fibrosis) with *pan*-HDACis results in an attenuation of fibrotic remodelling through the epigenetic rescue of antifibrotic genes and/or the action on fibrosis-related pathways as TGF-β signalling cascade [65]. Taking into consideration the chronic nature of PF, recent efforts focused on the evaluation of isoform-selective HDAC inhibition overexpressed in fibrotic lung tissue with the aim to avoid the toxic effects associated with long treatment with *pan*-HDACis. A selective HDAC3 inhibitor, namely RGFP966 (**5**, Figure 3), exerts its antifibrotic effect epigenetically, by downregulating the expression of fibrogenic proteins, inflammatory cytokines and Nrf2 antioxidant enzymes [67]. Furthermore, the selective spiroindoline-based HDAC6 inhibitor **6** (Figure 3) was able to revert the fibrotic phenotype, by reducing the mRNA expression for α-SMA, collagen types I and III, fibronectin, and HDAC6 in TGF-β1-stimulated lung tissue [23]. Compared to the other two isoforms, HDAC8 is mildly but significantly expressed in IPF lung tissue; thus, it was reasonable to elucidate the way in which its inhibition could impact fibrogenesis induced by TGF-β in normal human lung fibroblast (NHLFs). Saito and co-workers provided evidence that selective HDAC8 inhibition by NCC-170 (**7**, Figure 3) determines reduced contractility in TGF-β1-stimulated-NHLFs through the downregulation of α-SMA expression and cofilin dephosphorylation. Further studies suggested that HDAC8 inhibition by **7** leads to the increased H3K27 acetylation at enhancer regions of the antifibrotic mediator peroxisome proliferator-activated receptor-gamma (PPAR-γ), whose expression is promoted. Moreover, treatment with compound **7** led to a decrease in the fibrotic process in bleomycin-treated mouse lungs by repressing the expression of collagen type I and fibronectin [68]. Although it was shown that selective HDAC8 inhibition could ameliorate the pathogenesis of PF, target validation requires a further biological investigation concerning the mechanism behind the antifibrotic effect in scarring lung tissue. 

#### 3.2.2. Renal Fibrosis

Renal fibrosis occurs in acute kidney injury (AKI) and chronic kidney disease (CKD), in response to renal damage [69]. The fibrotic event is triggered by TGF-β1 that stimulates myofibroblasts differentiation, through the SMAD3 cascade, and promotes EMT by activation of STAT3 and β-catenin signalling pathways [70]. To date, the therapeutic plan is limited to palliative care; thus, recent efforts focused on the identification of new targets to counter and delay the progression of the disease [71]. The potential renoprotective and tissue reparative properties of HDACis in AKI and CKD were demonstrated in in vitro and in vivo models [60,61]. Among *pan*-HDACis, TSA (**3**) was largely studied: it can inhibit fibroblast differentiation and EMT by blocking STAT3 signals in the NRK49F rat kidney fibroblast cell line. In in vivo experiments performed on diabetic rats (STZ-induced diabetic kidney), **3** is able to revert fibrotic phenotype in the kidney, as demonstrated also in NRK52E—a rat kidney epithelial cell line. Recently, the role of HDAC8 in renal fibrosis has been highlighted, suggesting its potential as a target for AKI and CKD. Long and co-workers screened a series of known selective HDAC8is, possessing different scaffolds, in the zebrafish AKI (*zf*AKI) model. Notably, the treatment with PCI-34051 (**1**) and two tetrahydroisoquinoline (THIQ)-based hydroxamic acids led to phenotypic *zf*AKI efficacy at 4 μM. The compounds were also evaluated in human kidney organoids that can simulate some of the fibrotic events arising after AKI: among the three inhibitors, **1** exhibited an interesting ability in reducing collagen deposition. Encouraged by these results, compound **1** was also tested in a mouse model of AKI, showing a reduction of mRNA markers of renal fibrosis with no direct effect on the disorder [72]. Following these latter findings, previous work evidenced that HDAC8 inhibition with **1** could modulate the expression of profibrotic markers, attenuating renal fibrosis induced by unilateral ureteral obstruction in vivo and TGF-β1 exposure in vitro. The study allowed for demonstrating HDAC8 expression in renal tubular epithelial cells and its upregulation in a murine model, after UUO injury. As a result, the increasing deacetylase activity on cortactin, a non-histone HDAC8 substrate, implies overexpression of α-SMA, collagen type I, and fibronectin that can be suppressed by treatment with **1**. Moreover, HDAC8 inhibition mediates the dephosphorylation of SMAD3, STAT3, and β-catenin, which translates into the activation of the TGF-β1-related signalling pathways underlying fibrogenesis. PCI-34051 exerts its antifibrotic activity in renal epithelial cells either through the arrest of the cell cycle at the G2/M phase and the upregulation of the transcription factor Snail, two events that prevent EMT. As reported in the UUO kidney, selective HDAC8 inhibition re-established the level of expression of Klotho and BMP-7, two major reno-protective proteins [73]. 

Taken together, these results pinpoint HDAC8 inhibition as a new potential strategy to yield important beneficial effects in renal fibrotic diseases. 

#### 3.2.3. Liver Fibrosis

Liver fibrogenesis is an aberrant response to hepatic damage associated with diverse pathological conditions such as hepatitis virus infection, alcoholic and non-alcoholic fatty liver diseases. The key cellular mediators are the hepatic stellate cells which undergo phenotypic conversion into myofibroblasts after the activation of several profibrotic pathways, predominantly arbitrated by TGF-β. Once activated, they drive the fibrotic process through the overproduction of ECM proteins which is, in turn, enhanced by their irregular degradation from the metalloproteinases (MMPs). The effective pharmacological approach aimed to arrest the progression of liver fibrosis remains limited to the eradication of the main causative agent, while it is tempting to direct the research towards the development of new antifibrotic agents, regardless of their aetiology [61]. Given the considerable involvement of HDACs in fibrosis, their inhibition appeared to be a valuable strategy to reduce liver fibrosis [74]. Based on these observations, Park and co-workers identified HNHA (**8**, Figure 3), a *pan*-HDACi, as an antifibrotic modulator in hepatic tissue. The compound inhibits the activation and proliferation of PDGF-induced mouse primary HSCs and ameliorates liver fibrosis caused by bile duct ligation (BDL) in rats. It can exert multiple actions on different steps of fibrotic event, including the expression of profibrogenic effectors (α-SMA, TGF-β, collagen type I) [75]. Moreover, it was demonstrated that valproic acid (**9**, VPA, Figure 3), another *pan*-HDACi, prevents the activation of thioacetamide-treated-HSCs by promoting apoptosis and contributes to reducing the accumulation of ECM proteins via the enhancement of MMPs expression [76]. Furthermore, a deeper investigation led to confirm the epigenetic effect of VPA inhibition consisting additionally in modulating the expression of the miRNAs responsible of fibrogenesis [77]. Although there is much evidence that supports the beneficial effect of *pan*-HDACis in liver fibrosis, the research community moved on the identification of inhibitors endowed with a remarkable selectivity towards a specific isoform. Considering that HDAC8 is overexpressed in BDL mice, it was reasonable to hypothesize its correlation with hepatic fibrogenesis and, in turn, to verify it in cholestatic liver injury. The treatment of BDL mice with the selective HDAC8i SPA3014 (**10**, Figure 3) produced a diminished expression of collagen type I and α-SMA which derives from the induced suppression of TGF-β expression. These biochemical effects were supported by gross and histopathologic analysis that confirmed the capability of the HDAC8i to limit the shifting towards the fibrotic phenotype. In conformity with these in vivo data, SPA3014 decreases TGF-β1 expression and related downstream signalling pathways—MAPK-Smad2/3 and JAK2-STAT3—in LX-2 *h*HSCs, exerting a hepatoprotective effect. Furthermore, HDAC8 inhibition results in the upregulation of PPAR-γ which contributes to attaining the antifibrotic effect [78]. The target validation of HDAC8 for the treatment of liver fibrosis represents a considerable starting point to develop new agents whose therapeutic use can be declined in diverse chronic liver diseases. 

#### 3.2.4. Cardiac Fibrosis

Cardiac fibrosis is a pathological condition in which the main heart functions are compromised. In particular, atrial interstitial fibrosis affects the generation and conduction of electric signals from the sinoatrial node, driving the development of arrhythmia and thrombosis. Although mostly concomitant with cardiac hypertrophy, ventricular fibrosis is responsible for tissue stiffness and diastolic dysfunctionality [61]. In this context, HDACis displayed an interesting antifibrotic activity equally in atrial and ventricular fibrosis [79]. More in detail, TSA (**3**) revealed to be effective in attenuating atrial fibrosis and fibrillation when tested in HopX transgenic mice with left ventricular hypertrophy [80]. Another example concerns the use of VPA (**9**) to treat hypertensive rats. Compound **9** prevented cardiac hypertrophy and fibrosis by regulating the acetylation of mineralocorticoid receptors [81]. Several works that tried to elucidate the mechanism of action of *pan*-HDACis are coupled to as many attempts to clarify the contribution of the isoform-selective inhibition to the final antifibrotic effect. Recently, the role of HDAC8 was evaluated in cardiac hypertrophy and fibrosis using isoproterenol-induced cardiac hypertrophy model where the expression of HDAC8 is upregulated. The authors provided evidence that the mRNA levels of the fibrosis markers collagen type I, fibronectin, and CTGF diminish in response to the treatment with **1**, as well as the expression of α-SMA and TGF-β1 mRNA and related proteins. The mechanism by which the compound exerts its antifibrotic effects implies the inactivation of p38/MAPK, a downstream HDAC8 target involved in the onset and progression of cardiac fibrosis [82]. The inhibitor **1** was also selected to evaluate in which measure HDAC8 inhibition could impact transverse aortic constriction (TAC)-induced heart failure in mice. Western blotting analyses and q-RT-PCR have enlightened that the small molecule behaves as a negative regulator of fibrosis-related genes such as *COL1A1, FN1, ACTA2*, and *TGFΒ1* in TAC mice. Further examination has indicated that compound **1** can reverse the upregulation of TGF-β1 and phosphorylated Smad2/3, central key actors of fibrotic signalling, in vivo and in vitro TGF-β1-stimulated cardiac fibroblasts. The selective inhibition of HDAC8 mitigates cardiac fibrosis in two different disease models via overlapping mechanisms. These results allow for identifying HDAC8 as a potential pharmacological tool for the treatment of cardiac disorders accompanied by fibrotic state [83].

#### 3.2.5. Aberrant Wound Healing Associated with Diabetic Foot Ulcers (DFU)

Concomitantly with tissue remodeling, angiogenesis is a well-established process that occurs during wound healing, in the growing tissue. About 4% of diabetes mellitus patients present diabetic foot ulcers, multifactorial vascular complications that require lower limb amputation in the most severe cases References [84,85,86]. Many efforts have been focused on the identification of efficacious pharmacological approaches to treat different kinds of DFU that share impaired angiogenesis. In this frame, a recent work identified the LINC01435/YY1/HDAC8 pathway as a promising target for its impact on the angiogenetic process in diabetic wounds [87]. A preliminary study was performed to verify the effect induced by exosomes from high glucose-pretreated immortalized human epidermal cells (HG-Exos) in diabetic mice, whose wound repair improved after the treatment. Further investigations allowed for attributing this effect to exo-mediated uptake of LINC01435—A long noncoding RNA—that suppresses the migration and tube formation of human umbilical vein endothelial cells (HUVECs). Considering the role of the Notch pathway in angiogenesis, the authors extended the inspection of potential antiangiogenetic targets to various players engaged in Notch signalling, including the HDACs family [88,89,90]. This analysis highlighted the overexpression of HDAC8 in HUVECs, promoted by the nuclear translocation of the transcription factor YY1. Additional experiments highlighted that HDAC8 knockdown could enhance vessel formation and migration of HUVECs, enforcing the evidence that HDAC8/Notch signalling is involved in the antiangiogenetic effect of Exo-LINC01435. These promising results should encourage the research to exploit HDAC8 selective inhibition as a potential approach to promote wound healing in DFU.

### 3.3. Cancer

The term “cancer” encompasses a group of multifactorial diseases characterized by abnormal cell proliferation of the affected cells which might develop the capacity to invade other nearby sites or diffuse in other organs, generating metastatic tumours. Oncogenesis can occur via the activation of multiple pathways with the involvement of several targets, whose pharmacological regulation constitutes one of the most effective approaches against this disorder. Worldwide, metastatic cancers represent the second leading cause of death. Despite the considerable arsenal of available drugs, there is an urgent need for novel therapeutics due to their severe side effects often accompanied by drug resistance phenomena. Given that epigenetic modifications can contribute to the onset and progression of malignancies, the potential contribution of HDACs has been largely explored in different cancer types, leading to the FDA approval of *pan*-HDACis as anticancer drugs [91]. In this context, the search for new inhibitors endowed with enhanced isoform-selectivity appears more attractive and challenging, since this strategy might overcome the undesired effects deriving from treatment with broad-spectrum HDAC inhibitors. Among the isoforms under investigation, HDAC8 has been proven to interfere with tumorigenesis. Given that HDAC8 plays a pivotal role for p53 expression, its enzymatic inhibition showed to be effective in tumour cells harbouring a p53 mutation. Nevertheless, further studies proved that HDAC8is can exert their antitumoural action through the activation of alternative mechanisms. Several reviews elaborated on the beneficial effect deriving from HDAC8 inhibition in a wide range of cancers taking place in blood, breast, liver, colon, lungs, and nervous system [10,27,92,93]. In this section, we give an overview of the most recent advances relative to HDAC8 engagement in some of the mentioned cancer types clustered in haematological and solid tumours.

#### 3.3.1. Haematological Malignancies 

Acute myeloid leukaemia (AML) can be classified as a heterogeneous series of malignant disorders featuring aberrant proliferation and differentiation of myeloid cell lines in the bone marrow. Based on the early discovered antiproliferative properties of HDAC8is against malignancies, further investigations were carried out to deeply comprehend their mechanism of action. Recently, Spreafico and co-workers demonstrated that treatment of AML cell lines (OCI-AML5, PLB985, THP-1, and AML193) with **1** restrained cell proliferation, inducing the cell cycle arrest along with apoptosis via activation of p53 signalling. This result was comparable with those obtained from the experiment conducted in the zebrafish disease model overexpressing HDAC8. Since p53-null HL60 is also sensitive to HDAC8 inhibition by **1** at 50 μM, some efforts were requested to identify the alternative mechanism underlying the cycle arrest in G0/G1 phase. The outcomes of the experiments indicated that HDAC8 inhibition determined the activation of the canonical Wnt pathway, whose dysregulation is associated with AML [94].

The potential involvement of HDAC8 was also explored in mantle cell lymphoma (MCL), a severe non-Hodgkin lymphoma that requires the use of high-dosed antitumour drugs with a negative impact on the therapeutic index. The selective inhibition of HDAC8 can induce caspase-dependent apoptosis in MCL cell line resulting in a cytostatic and cytotoxic effect. In contrast to *pan*-HDACis, inhibition induced by compound **1** did not impair the viability of natural killer cells but preserved their functional response to cancer conditions [95].

#### 3.3.2. Solid Tumours

A growing body of work has pinpointed epigenetic modulation as a valuable strategy to tackle breast cancer. In the research of new epigenetic targets, HDAC8 exhibited antiproliferative properties in different breast cancer subtypes. Chiu et al. provided evidence that HMC (**11**, Figure 3), a selective HDAC8i, was able to cause caspase-dependent apoptosis in the MCF-7 cell line through the suppression of Akt/mTOR signalling and the activation of PPAR-γ pathway, coupled with DNA damage induced by ROS production. In addition, **11** stimulated autophagy, an explicit survival mechanism that enhances cell growth inhibition in the treated MCF-7 cells [37]. Further investigations have enlightened that HDAC8 promoted dissemination of breast cancer cells in vitro and in vivo, through the activation of EMT, a central process underlying metastasis. Enzymatic inhibition with **1** preserved protein stability of Snail, a fundamental modulator of EMT, via other two concomitant and correlated mechanisms: the increase in GSK-3β phosphorylation which is, in turn, assisted by HDAC8-regulated AKT phosphorylation. This finding represents a good starting point to further elaborate on the therapeutic effect of HDAC8i for severe and metastasized breast cancer [96]. 

Consistent with this latter work, it was proven that treatment with **1**, as monotherapy or in combination with cyclophosphamide, adriamycin, and 5-fluorouracil (CAF), impaired tumour cell survival in basal-like breast cancer, by suppressing some transcription factors involved in the EMT process (Gata3, Elf5, Rora and Grhl2). The effect was evaluated in murine and human basal-like breast cancer cell lines—pG-2 and HCC1806, respectively [93]. Experiments conducted in HeLa cervical cancer cells could attribute the antiproliferative effect to the downregulation of HDAC8 activity. Treatment with inhibitor **1** decreased the level of the acetylated α-tubulin (Ac-Tub), which plays an important role in cell migration and in G2/M phase of the cell cycle. As a final effect, reduction of mitosis and a diminished capacity of dissemination were observed [97].

Many examples in the literature reported the use of HDACis to empower the immune checkpoint blockade in cancer by restoring or enhancing the capability of T-cells in detecting and destroying the tumour cells. Furthermore, the immunomodulatory properties of HDACis were exploited to potentiate the action of immune checkpoint inhibitors, leading to a synergistic antitumour effect [98,99,100,101,102,103,104,105,106]. 

Recently, it was discovered that acetylation of H3K27, induced by selective HDAC8 inhibition, entails the secretion of T cell–recruiting chemokines by hepatocellular carcinoma cells, resulting in enhanced CD8^+^ T cells infiltration in preclinical disease model [107].

Furthermore, it was found that the selective HDAC8i **1** could impair the viability and the migration of human and murine glioma cells, and in agreement with these findings, it was proven to reduce the tumour size in mice models of glioma. The effect is ascribable to the increase in the Ac-Tub, as seen in HeLa cells. In addition, HDAC8 inhibition enforces the immune response mediated by natural killer cells through positive regulation of the transcription of ligands for NKG2D receptor, whose activation is responsible of NK cytotoxicity in tumour cells [108]. The anticancer properties of **1** have been exploited in glioblastoma multiforme (GBM), with the aim to overcome the adverse effect of the chemotherapy with temozolomide (TMZ). This drug induces an increase in O^6^-methyl-guanine DNA methyltransferase (MGMT) which acts in the repair of DNA damage. Compound **1** allowed reversing this drug response in TMZ-resistant cells, by cooperating with ADRM1, the proteasome receptor. Therefore, HDAC8 inhibition determines reduced viability in GMB cell line due to the arrest of the cell cycle, enhanced by a failed repair of DNA damage [109]. 

Neuroblastoma is a typical childhood tumour of the peripheral nervous system which has drawn the attention of the research community because of its high lethality rate. Among the screened targets, in vitro and in in vivo studies revealed that HDAC8 is involved in this pathology. HDAC8 inhibition is correlated to the block of cell proliferation, the induction of cell cycle arrest and the promotion of differentiation in neuroblastoma cell line [110]. Recently, further studies have strengthened the idea that HDAC8is could represent potential therapeutic tools to reverse the disorder, especially when their action is potentiated by other factors, which are discussed in the next paragraphs [111,112].

## 4. Neuropathological Disorders and Conditions

In recent years, the therapeutic potential of HDACis was examined in different neurological conditions that differ from the malignant and X-linked disorders. Psychiatric disorders, neurodegenerative diseases, and other comorbid neurological conditions appear to be correlated to impaired homeostasis of histone acetylation, and, accordingly, the re-establishment of the normal HDACs activity was proven to exert a neuroprotective effect [10]. Neuroprotection derives from the targeting of histones along with other non-histone substrates such as the heat shock protein 70 (HSP70), brain-derived neurotrophic factor (BDNF), and glucose regulated protein 78 (GRP78) [113,114,115,116,117]. Their dysregulation is associated with the initiation and progression of various neurological disorders. As illustrated below, the effect of HDACis was evaluated in diverse neurodegenerative disease models where an improved neuronal survival was attained through the activation of several mechanisms.

Parkinson’s disease is a neurodegenerative syndrome triggered by the progressive loss of dopaminergic neurons in the substantia nigra. The *pan*-inhibitor VPA (**9**) was shown able to retrieve the toxic action of 6-hydroxydopamine in SH-SY5Y dopaminergic neuronal cells, via the downregulation of the apoptotic caspases 3, 7, and 9. Nevertheless, literature data suggest that, generally, HDACis can behave as promoters or inhibitors of Parkinson’s disease pathogenesis, reasonably depending on the epigenetic status and the tissue specificity [118].

Amyotrophic lateral sclerosis is a neurodegenerative disorder that determines progressive damage to motor neurons of the central nervous system (CNS), which result in muscle atrophy and death. The application of HDACis in amyotrophic lateral sclerosis model provided promising results. Among them, a significant achievement is represented by the effect of VPA (**9**) in reducing the death of motor neurons in G86R SOD1 mutant mice [119].

Multiple sclerosis is a demyelinating disease that impacts the brain and the spinal cord. Recently, a relevant increase in lysine acetylation in myelin protein was described for an autoimmune encephalomyelitis model of multiple sclerosis [120]. Previously, it was reported that TSA (**3**) treatment ameliorated inflammation in the spinal cord and prevented demyelination and axonal loss in encephalomyelitis mice. Furthermore, the inhibitor was able to enhance the activity of neuroprotective proteins and decrease the expression of proapoptotic factors [121].

Alzheimer’s disease is a neurodegenerative disorder, featured by memory loss accompanied by cognitive and behavioural impairment which can progress in dementia. The beneficial effect of *pan*-HDACis in Alzheimer’s disease pathogenesis encouraged the research to evaluate the contribution of the single isoforms. Among them, HDAC6 was revealed to be involved in the learning impairment and loss of memory in Alzheimer’s disease mouse model [122].

In this frame, there is limited information about the enrolment of HDAC8 in the mentioned neuropathological diseases, probably due to its low level of expression in the brain [123]. However, it was proven, even in other disorders, that the overexpression of the target does not always represent a necessary condition to exploit its modulation for therapeutic purposes.

In a recent study, the effect of the selective HDAC8 inhibitor WK2-16 (**12**, Figure 3) was evaluated in striatal lipopolysaccharide (LPS)-induced neuroinflammation in C57BL/6 mice. The treatment (30 mg/kg) with **12** alleviated the neuroinflammation and ameliorated the neurological function, via the suppression of astrocyte and microglia activation. Experiments conducted in microglial BV-2 cells confirmed the ability of compound **12** to induce concentration-dependently microglia inactivation (0.5, 1, and 2 μM). Moreover, the impact of HDAC8 inhibition on proinflammatory markers was assessed in LPS-activated BV-2 microglia: the results displayed a downregulated expression of cyclooxygenase-2 (COX-2) and inducible nitric oxide synthase (iNOS) coupled with reduced production of TNF-α. Mechanistically, the effect is ascribable to the activation of STAT1/-3 and Akt signalling pathways. Given that neuroinflammation and microglia activation play a key role in neurodegenerative conditions and brain abscess, these achievements indicate HDAC8 as a promising target for these disorders [124]. The anti-inflammatory effect deriving from HDAC8 inhibition was explored by Hendrix and co-workers in a spinal cord injury (SCI) model. This disease occurs after a traumatic insult that leads to neuroinflammation, oedema, and neuronal cell death. Although the results showed a decrease in infiltration of ionized calcium-binding adaptor molecule-1 positive macrophages (Iba-1^+^) after SCI, no functional recovery was observed [125]. Thus, further investigation should be carried out to elucidate this outcome. 

Recently, Katayama et al. studied the embryoid body formation in HDAC8i-treated-P19 cells, with the aim of elucidating the HDAC8 function in neurodevelopment. The selected NCC-149 (**13**, Figure 3) inhibitor downregulated the expression of the neuronal marker NeuN and induced the formation of smaller embryoid bodies in comparison with the non-treated cells. Furthermore, the selective HDAC8 inhibition blocked the cell cycle at G2/M phase and decreases the expression of cyclin A2 and cyclin B1 genes. Taken together, these data propose HDAC8 as a regulator of neural differentiation, a key process in neurodevelopmental process [126].

The reported studies thus served as a proof-of-concept that HDAC8 inhibition might offer novel therapeutic options for neurological disorders.

### 4.1. HDACis Drug Design

Over the past few decades, the target validation of HDACs for a mounting array of diseases has received a great attention by medicinal chemists who have addressed many endeavours to the development of novel HDACis. The availability of the enzyme crystal structures enabled for the study of the environment of the active site at a molecular level and, subsequently, to hypothesize the key binding interactions [20]. Structure-based rational design of potential HDAC inhibitors relies on co-crystal structures which represent the main source of information concerning the target-small molecule interactions. Together with computational and structure-activity relationship (SAR) studies, structural data guided the identification of the three main pharmacophoric elements common to HDACis: (i) the cap group, a hydrophobic moiety that recognizes and interacts with the rim surface of the catalytic tunnel; (ii) the zinc-binding group (ZBG) able to tightly chelate the Zn^2+^ ion located in the active site; (iii) the linker consisting in a hydrophobic spacer (HS) that links the cap and the ZBG trough a polar connecting unit (CU), ensuring the proper accommodation of the molecule in the catalytic domain [127,128]. The identification of a common pharmacophore has driven the discovery of potent *pan*-HDACis, several of which were still under investigation in clinical trials; meanwhile, five have already attained FDA approval [129,130]. 

The nature and the geometry of the intermolecular interactions between the three moieties and the active site are the main factors that govern the affinity and selectivity towards a specific isoform. Thus, the drug design of selective inhibitors is mainly based on the proper modifications of the three pharmacophoric elements which should allow the compounds to discriminate among the isozymes [131].

Several approaches were applied to enrich the chemodiversity of HDACis, mostly based on the different cap groups identified by scaffold hopping, repurposing of privileged cores, or screening of natural products [20,132].

Generally, HDACis presents a hydroxamic acid as ZBG since it confers a high affinity to the compounds towards the target due to its extraordinary capacity of zinc coordination in a bidentate fashion. However, the rising evidence of its potential mutagenicity, and poor bioavailability, prompted the validation of new ZBGs such as anilide, thiol—mostly masked as a disulphide-, azetidin-2-one, some of which preferentially bind to specific HDAC isoforms [92]. Regarding the spacer, there are a wide collection of linkers able to impart the best molecular orientation inside the catalytic pocket. In this context, a systematic analysis concerning the connection points between HS and the other two moieties could provide a guideline about the principal parameters to consider in the choice of a suitable linker, such as its polarity, length, and rigidity [127]. 

Even though the design of HDACis prevalently relies on the three-motif model, many examples reported in the literature do not completely adhere to it and that is closely correlated with the isotype taken into account [133]. 

### 4.2. HDAC8 Drug Design

**Figure 4:**
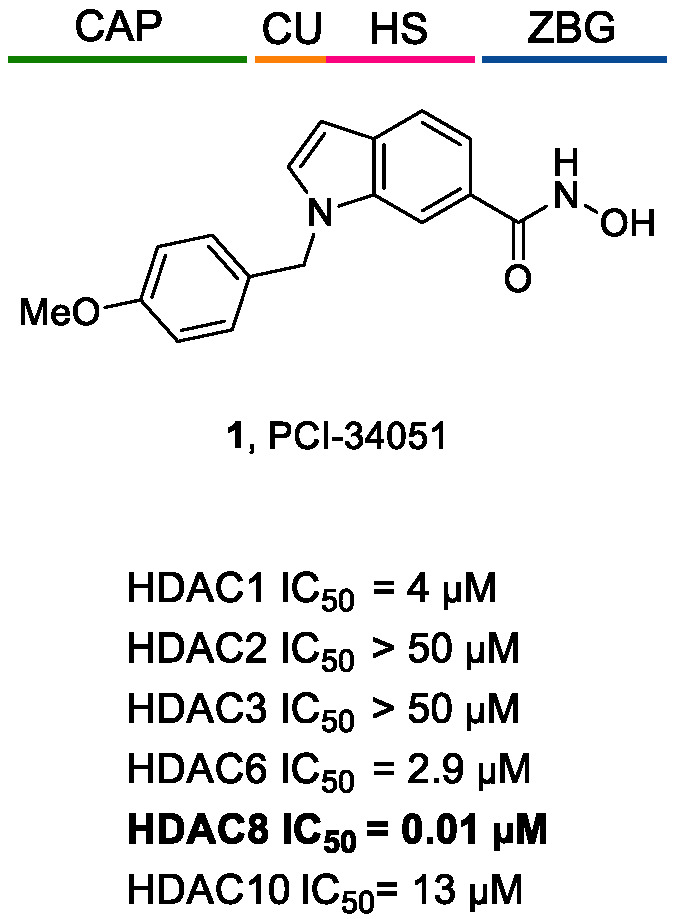
Activity and selectivity profile of PCI-34051 (**1**) [19].

In light of the advantages that could derive from the isotype-selective targeting, computational tools were employed to extend the information concerning the specific binding interaction between the active site and the known selective HDAC8is [134]. Due to the flexibility of the active site residues and loops, especially the L1 and L2, computational chemists found it hard to assess the biologically favoured conformation that stabilizes the inhibitor in complex with the enzyme [18]. However, many studies highlighted that the selective HDAC8 inhibition is ascribable to the preferential binding of the inhibitors to a unique HDAC8 sub-pocket which allows the compound to discriminate among the other isoforms, including those belonging to class I [135]. The catalytic tyrosine in L7 loop, together with some aminoacidic residues at L6 and L1 loops, create the specific HDAC8 pocket, a shallow groove where the selective inhibitor can lie in their L-shaped conformation. The capability of the compound to adopt a low-energy L-shape ensures selective interactions with the active site, since this conformation was proven to be complementary with the topology of the catalytic pocket (Figure 2 and Figure 4). Furthermore, the interactions between the catalytic tyrosine and the ZBG stabilize the complex by reducing the high flexibility of the L7 loop. Notably, bulkier HDAC8is as compound **13** can be involved in additional contacts with the sub-pocket at its backside. Even though the catalytic tyrosine is conserved in class I and IIb HDACs, the amino acid residue is partially covered by a larger L1 loop, which cooperates with L6 loop to form a lock over the sub-pocket. As a result, the active site appears sterically different in comparison with HDAC8, preventing HDAC8is from binding to the isozymes 1, 2, 3, 6 and 10. In addition, the selectivity over class IIa HDACs is guaranteed by the replacement of tyrosine with histidine which, in turn, forms another specific pocket. These findings emphasized that selective HDAC8 inhibition could be achieved by properly analysing the potential conformations that compounds can adopt in the catalytic site, given that small structural modifications might affect the binding interactions, and consequently, the affinity and the selectivity towards a specific HDAC isoform [18].

## 5. HDAC8 Inhibitors

The research of selective small molecule HDAC8is is still a challenging task in the field of medicinal chemistry. To date, several inhibitors have been identified, but only few of them have been evaluated in in vitro and in vivo experiments [130]. Supplementary studies are required to refine the approaches pursued for the drug design. To attain this goal, a classification of HDAC8is might be useful to pinpoint the common features which could be exploited for the development of novel potent and selective compounds. In this frame, we will provide a snapshot of the currently known HDAC8is, corroborated by thorough SAR studies centred on the analysis of the key pharmacophoric elements. Taking into account the chemodiversity of the cap groups employed in HDAC8is, we found it more convenient to categorize the inhibitors on the basis of (i) different ZBGs, distinguishing between hydroxamic acid motif and other novel ZBGs, and (ii) conserved linkers driving the molecules to adopt the suitable orientation. Profiling of the inhibitors was supported by inhibitory potency data deriving from the enzymatic assay performed on HDAC8 and other representative isoforms. Although the selectivity should be proved on the entire panel of HDACs, evidence allowed for narrowing-down the choice of the isoforms to be included in the enzymatic test to those sharing closer similarities with HDAC8. Specifically, it is important to consider HDAC1-3, belonging to class I, and HDAC6 (class II), whose similar structural features compromise the discrimination between the two targets (see also Figure 2). Based on these observations, biochemical assays might guide the identification of the hit compounds which should maintain a good selectivity index (SI) (that, for attaining cellular selectivity, should be above 30) at least towards the representative class I isoforms and HDAC6. Hit validation should rely on proof of target engagement in cell-based studies evaluating the level of acetylation of SMC3 and H3, preferential HDAC8 targets, rather than Ac-Tub, typical marker of HDAC6 inhibition. However, depending on the expression level of HDACs and the molecular patterns involved in the cell lines under investigation, further considerations are required to define the real selectivity towards the HDAC8 isoform [20].

### 5.1. HDAC8is Bearing Hydroxamic Acids as ZBG

#### 5.1.1. Aromatic-Based Linkers

In the search of novel HDAC8is, one of the most critical parameters to consider is the rigidity of the linker, the moiety connecting the cap group to the ZBG motif. To date, compounds containing an aromatic-based linker tend to display higher potency—in the nanomolar range—than those having a more flexible one. 

**Figure 5:**
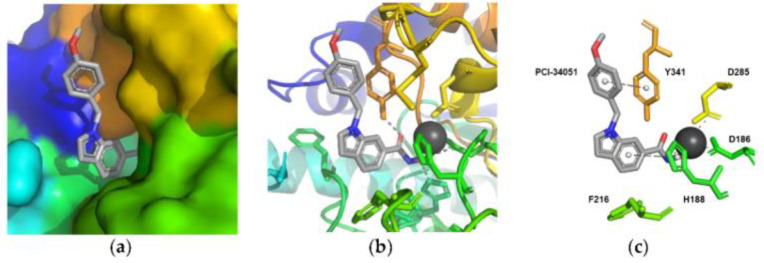
Representation of the crystal structure of mutant *sm*HDAC8-H292M in complex with compound **1** (PDB code: 6HSF): close-up view of the HDAC8-specific pocket shown as (**a**) surface representation (**b**) ribbon and stick (**c**) schematic binding interactions with the principal residues. The crystal structure was downloaded from the Protein Data Bank, visualized by Pymol, (**a**,**b**), and analysed for protein-ligand interactions profiles using PLIP online server (**c**) [138]. PCI-34051 (**1**) is depicted in light grey.

Among the first group of inhibitors, PCI-34051(**1**, Figure 2, Figure 3, Figure 4 and Figure 5) is the most widely employed for the target validation in different diseases, due to its considerable isoform-selectivity demonstrated by computational, crystallographic, and cellular studies. The compound displayed a nanomolar inhibitory potency against *h*HDAC8 (IC_50_ = 10 nM) along with >200-fold selectivity over isozymes 1 and 6, and > 1000-fold selectivity over isozymes 2, 3 and 10 (Figure 4) [19]. Compound **1** was rationally designed according to the three-motif pharmacophoric model of HDACis: the compound presents an indole core which serves as hydrophobic spacer between the hydroxamic acid and the methoxyphenyl cap group, conferring a suitable rigidity to the structure.

In recent years, many attempts were accomplished to produce well-diffracting crystals of *h*HDAC8 in complex with **1** and, in parallel, a mounting body of evidence suggested the reliability of the binding information deriving from soaking experiments of apo *sm*HDAC8 with HDAC8is [136]. Given that, co-crystallization experiments of *sm*HDAC8 with **1** were performed, leading to high-resolution complex structures (Figure 5, PDB code: 6HSF). The analysis of the results revealed that the cap moiety is perpendicularly oriented over the side chain of the catalytic tyrosine of *sm*HDAC8 Y341 (*h*HDAC8 Y306), with which forms T-shaped π−π stacking interactions. To translate this result to *h*HDAC8, differences in the environment of the active site were overcome by conducting the same experiments in “humanized” *sm*HDAC8-H292M mutant. In addition, in this case, the results highlighted that the small molecule is involved in close nonpolar contacts with Y341, adopting an L-shaped conformation which ensures the binding with the unique HDAC8 sub-pocket [18]. Despite a series of analogues being investigated, biological and cellular studies confirmed **1** as the best performing candidate to be used for further studies concerning HDAC8 inhibition [137]. However, **1** remains a useful tool to investigate the role of HDAC8 in several aberrant pathways, without any other application in clinical studies, due to its low molecular weight and solubility in physiologic media.

In 2011, three potent HDAC8is were developed by applying a well-established synthetic method in 96-well plates which were directly employed in a high-throughput screening biochemical assay [139]. Aiming to perform high-yielding reactions, the authors selected building blocks bearing hydrazide or aldehyde for their high reactivity, while hydroxamic acid-based substrates were included to introduce an entrenched ZBG in the final compounds. The synthesized compounds were tested in enzymatic assays against *h*HDAC2, *h*HDAC3, and *h*HDAC8, allowing for the identification of three inhibitors, **14**, **15**, and **16**, endowed with high selectivity towards isoform 8 (Table 1). The compounds are structurally related since they present a hydroxamic acid moiety directly attached to a phenyl spacer which, in turn, bears a hydrazide as a connecting unit (CU). Although their cap groups showed different bulkiness and rigidity, the compounds maintained comparable activity/selectivity profiles. Further pharmacodynamic studies should be carried out to better understand the binding mode of these inhibitors in the HDAC8 active site. Although the crucial information about the selectivity for HDAC6 is still not available, it is reasonable to attribute their high selectivity to the *meta*-substitution of the ZBG motif in the phenyl ring that could impart an L-shaped conformation, complementary with the topology of the catalytic domain [139]. It was found that HDAC6 better tolerates inhibitors with *ortho* than *para*-substituted aromatic linkers since they can lie in the wider channel created by L6 and L1 loops, forming additional contacts with the active site [20,140].

Among the fragment-based assembly methods, click chemistry was revealed as an efficient and versatile approach to develop extensive libraries of compounds with a straightforward synthetic protocol. This strategy was exploited to prepare a library of 120 potential HDACis candidates, whose synthesis and screening with enzymatic assay were performed in microtiter plates. The selection of the building blocks was, as usual, accomplished according to the pharmacophore model of HDACis; thus, each compound contains a ZBG, a cap group and a triazole-based linker. The screening identified two potent HDAC8 inhibitors, **17** and **18** (Table 2), which inspired a novel 31-member library of analogues [141]. From this process of hit optimization, compounds **19** and **13** emerged as new potent and selective HDAC8 inhibitors over the isoforms 1, 2, 4 and 6. Their structures share the same ZBG (hydroxamic acid) and linker (phenyl spacer with triazole as polar CU), while differing for their cap group: in particular, the homologation of the benzyl group of **17** with methylene or an aromatic sulphide group led to **18** and **13**, respectively, while a 3-thienyl group was used in **19** (Table 2). 

Each compound displayed a high potency in the same nanomolar range and similar selectivity profile, which is less pronounced for HDAC6 (which is the main issue). A molecular modelling study suggested that the triazole ring might favour **13** to adopt a U-shaped conformation in the active site, fixing the orientation of the phenylthiomethyl group in the hydrophobic sub-pocket. The selectivity was demonstrated either by cell-based studies which allowed for proving the target engagement and the antiproliferative activity of **13** in T-cell lymphoma cells [141]. 

In 2014, the same research group investigated the substitution of the triazole with the phenyl group and bioisosteric replacement with thiazole, oxadiazole, reversed triazole and thiophene. Five new analogues were synthesized and analysed for their inhibitory activity towards HDACs from nuclear extract and the single isozymes 1, 2, 3, 4, 6 and 8. The enzymatic assay demonstrated that the potency and the selectivity towards HDAC8 is maintained for the compounds containing the five-membered aromatic ring, which favours a suitable conformation for the binding to the active site. Among the compounds, the reversed triazole derivative **20** (Table 2) was proven to be the most potent HDAC8 inhibitor, also when compared with **13**, while cellular studies enlightened that compound **7** (**NCC-170**, Table 2) is the most selective and the most potent against T-cell lymphoma cells [142].

Inspired by potent and selective inhibitors against *sm*HDAC8, Heimburg and co-workers developed three series of benzhydroxamic acids, properly functionalized at *meta* position with amino, phenylcarbamoyl or benzyloxy moieties for favouring the L-shaped conformation [143]. The enzymatic assay revealed compound **21** as the best candidate of the first series, owing to its enhanced inhibitory potency against HDAC8 (IC_50_ = 35 ± 4 nM) coupled with a SI of 534 and 411 over HDAC1 and HDAC6, respectively (Table 3). However, compound **22** exhibited the most enhanced selectivity in BE(2)-C cells, since it was able to stronger upregulate the acetylation of SMC3, a bona fide substrate of HDAC8. This latter inhibitor presents a 4-methoxy phenyl spacer which is connected to the benzylic cap through an aniline-based polar unit whose conversion in ether led to compound **23** (Table 3). The promising activity/selectivity profile of **23** (HDAC8 IC_50_ = 35 ± 4 nM, HDAC1 IC_50_ = 12.1 ± 5.7 μM, SI = 448; HDAC6 IC_50_ = 2.9 ± 0.3 μM, SI = 107) and **22** was investigated in cellular studies (Table 3). Treatment with the two HDAC8is showed the target engagement at cellular level which was exploited to evaluate their anticancer activity in neuroblastoma cells. In particular, the compounds were demonstrated to be effective in upregulating the outgrowth of neurofilament positive neurite-like structures and the marker genes of differentiation, countering the process of tumorigenesis [143].

Collectively, these latter results suggest that *meta*-substitution in benzhydroxamic acid ensures to discriminate HDAC8 among the other isozymes, especially HDAC6 with which shares more structural similarities in the active site. In light of these findings, a series of novel *N*-hydroxy-3-sulfamoylbenzamide-base selective HDAC8is was developed: according to the pharmacophore model, each compound contains a hydroxamic acid as ZBG and a phenyl spacer bearing a sulfamoyl moiety (CU) directly tethered to different cap groups. Among the derivatives, **24**, **25**, and **26** displayed a potent inhibition towards HDAC8 and a significant selectivity over the isoforms 2 (SI = > 180) and 6 (SI = ~30), which was proven by Western blot analysis (Table 4) [34]. These data were rationalized by molecular docking studies that highlighted the key role of the sulfamoyl motif in facilitating the interaction of the cap groups with the HDAC8 sub-pocket. Moreover, the three inhibitors exhibited an antiproliferative activity to T-cell leukaemia cell lines Jurkat, Molt-4, and neuroblastoma cell line SK-N-BE-(2) [34].

Recently, rigid L-shaped scaffolds were evaluated to achieve the selectivity towards HDAC8, and among the strategies explored for this purpose, it was found fruitful to move from *meta* to *para* position of ZBG in a phenyl spacer. The suitable structural rigidity was attained in a class of constrained benzanilide derivatives which were rationally designed to selectively target HDAC8. Aiming to draw a preliminary SAR, 3-mono or 3,5-disubstituted cap groups (R_1_) were combined with a benzhydroxamic moiety through conformationally rigid CUs, ranging from amide, sulfonamide and methylene motif. In the enzymatic assays, benzanilide-based compounds gave the best results. Of note, the comparison between the compounds **27** and **28** (Table 5) in terms of inhibitory potency and selectivity led to the hypothesis that the alkyl substitution of the amide could favour the E (or L-shaped) conformation, contrary to proton substituent which allows the molecule to adopt Z conformation [24]. Starting from this observation, the hit optimization provided the incorporation of an isopropyl or methylene cyclopropyl group on the most active ad selective compounds resulting in **29**, **30** and **31** (Table 5). 

These analogues were identified as the best candidates to further cellular investigation, owing to their potent HDAC8 inhibitory activity (IC_50_ = 66, 23, and 66 nM) and up to 410-fold selectivity for HDAC8 over the other HDACs. Treatment of neuroblastoma cell (BE(2)-C) with **29** and **31** highlighted the target engagement together with their potential use as chemotherapeutics and biological probes [24].

#### 5.1.2. Flexible Aliphatic-Based Linkers

**Figure 6:**
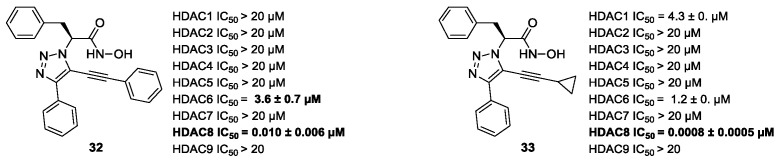
Structure and IC_50_ of **32** and **33** [144].

Several HDACis display flexible aliphatic linkers able to appropriately orientate the ZBG and the cap in the active site. Although the binding mode of the known HDAC8is requires a suitable scaffold rigidity to ensure the access to the HDAC8-specific pocket, few examples of inhibitors possessing a slender spacer are reported in literature. However, different endeavours focused on the evaluation of the best balance between the length and the flexibility of the linker in accordance with the topology and conformation of the catalytic domain. In 2016, a research group attempted to identify HDACis by repurposing of available libraries of structurally different compounds which were appropriately modified through the introduction of a hydroxamic acid motif. The screening campaign evaluated the inhibitory activity against a panel of HDACs (1–9); among the 120 compounds (hydroxamic acids and methyl hydroxamates), only **32** (Figure 6) exhibited a marked potency and selectivity towards HDAC8 [144]. In order to perform a SAR analysis, different analogues were prepared to assess the importance of the hydroxamic acid motif, the stereochemistry and the substitution of the triazole for HDAC8 inhibition potency. However, all the compounds were shown to be inactive against HDAC8; therefore, a novel array of derivatives was designed, modifying the side chain of the amino acids and utilizing alternative substituents in the triazole core. The results obtained from the enzymatic assay drew a preliminary SAR which could be articulated in four essential structural features: (i) *S*-stereochemistry, (ii) a benzyl moiety in the α-carbon to the ZBG, (iii) a 3-substituted triazole with an acetylene moiety functionalized with small groups, and (iv) a small aromatic ring, without linkers at C4 of the triazole. The most active HDAC8i **OJI-1** (**33**, Figure 6) was assayed for its selectivity over isozymes 1-9, showing to be a valid tool to study HDAC8 inhibition in different disease models [144].

The availability of structural and conformational information of HDAC8 enabled the exploitation of a computer-aided molecular design approach to develop a series of potential selective inhibitors of C1-substituted tetrahydroisoquinolines (THIQs). In a preliminary study, a set of amine-based derivatives was prepared with the aim of assessing the effect of the linker length on the HDAC inhibitory potency. Their general structure presents a benzylic amine functionalized with a second benzyl moiety or ethyl group and a carbon chain bearing the hydroxamic acid. Each compound exhibited micromolar activity towards the isoform 1, 2, 3, and 8, but it was interesting to denote and exploit the considerable HDAC8 selectivity of **34** over the other isoforms (Table 6) [145]. Therefore, molecular docking studies were performed, and the visualization of the results allowed for rationalizing the design of new cyclic analogues containing a further linker bridging the two aromatic moieties in **34**. The resulting C1-substituted THIQs were tested in the enzymatic assay against the mentioned HDACs. The improvement of the potency was attained in **35** and **36** which displayed IC_50_ values of 82 and 55 nM, respectively (Table 6); the compounds maintained the selectivity over the other class I HDACs, while the inhibition of HDAC6 was about 50% at 10 μM. In silico studies suggested that the C1-THIQ scaffold, serving as cap group, is able to make hydrophobic contacts with the HDAC8 pocket, due to the suitable orientation of the ZBG in the active site imparted by the ethyl spacer. The inhibitors were subjected to a pharmacological characterization in neuroblastoma cell line, where **36** was able to induce a concentration-dependent cytotoxicity similar to or better than that of PCI-34051 [145]. 

Recently, Trivedi and co-workers employed alkylpiperidine and alkylpiperazine linker moieties in a new class of hydroxamate derivatives possessing an interesting activity/selectivity profile against HDAC8 [146]. The drug design provided the evaluation of the length of the side chain attached to the ZBG and the screening of cap groups endowed with different hydrophobicity and bulkiness. The results obtained by the enzymatic assay revealed that HDAC8 inhibition is favoured by the presence of a bulky heteroaromatic cap, combined through a urea bound (CU) to the piperazine linker containing a three-methylene chain. Differences in activity between piperidine and piperazine-based compounds can be ascribed to the additional contacts that urea can make in the enzyme pocket with respect to the amide bound. SAR analysis, assisted by molecular docking, pointed out **37**, **38** and **39** as the most selective HDAC8is of the series, having SI HDAC3/HDAC8 values of 17, 16, 13, respectively (Table 7). 

Despite their micromolar activity, the inhibitors exhibited interesting cytotoxicity activity at 7-12 μM in human solid tumour cell lines along with the leukaemia cell line (A-549, HeLa, MCF-7, B16F10, Jurkat E6), owing to their proapoptotic effect demonstrated by caspase3/7 assay [146].

#### 5.1.3. Alkenyl-Based Linkers

The exploration of an intermediate structural rigidity between those of the aromatic and the aliphatic linkers led to a limited number of HDAC8is containing alkenyl hydroxamic acid moiety. Nevertheless, it was found that cinnamon extract and its bioactives can behave as negative modulators of HDAC8, leading to consider the employment of their structure in the drug design of novel active small molecules [147]. In 2012, Huang et al. developed two set of phenyl-*N*-hydroxycinnamides as potential HDAC8is, by combining pharmacophoric features of known HDACis [148]. The evaluation of their enzymatic inhibition by biochemical assays revealed that the *ortho*-substituted phenyl *N*-hydroxycinnamides were more active than the *para*-substituted analogues towards HDAC8 isoform with respect to HDACs from HeLa nuclear extract. This result suggested that the *ortho* substitution allowed the compounds to selectively bind to the catalytic HDAC8-specific sub-pocket, as confirmed by molecular docking studies. To this end, a sub-class of analogues was prepared by incorporating different linker chains added to the *ortho*-aryl groups. Their screening by enzymatic assay proposed **40** (Table 8) with no flexible linker as the best performing compound against HDAC8 (IC_50_ = 724 nM); thus, other aromatic moieties were explored in six novel analogues. The structure-guided design led to the identification of **41** and **12** (Table 8) as potent HDAC8is displaying IC_50_ values of 5.7 ± 0.1 nM and 27.2 ± 3.1 nM, respectively. The compounds were demonstrated to be selective over a panel of HDACs (isoform 1-4, 6, 10, 11); thus, their potential antiproliferative properties were evaluated for various human lung cancer cell lines (A549, H1299, and CL1-5) where HDAC8 is differently overexpressed. Consistent with this, the results suggested the potential use of **12** as a pharmacological tool to investigate the engagement of HDAC8 in tumorigenesis [148]. 

In 2015, a series of *ortho*-substituted *N*-hydroxycinnamides were patented for AML [149]. Among them, **11** (Figure 1) was employed for target validation in breast cancer. The compound is structurally related to **12**, since it contains an alkenyl-based spacer directly connected to a hydrophobic and rigid cap which can occupy the hydrophobic HDAC8 sub-pocket [37].

**Table 8:** General structure and IC_50_ values of **40**, **41**, and **12** [148].

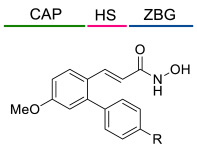			
IC_50_ (μM) ^2^	40 R_1_ = H-	41 R_1_ = Br-	12 R_1_ = Ph-
HeLa HDACs ^1^	>10	>10	>10
*h*HDAC1	ND	4.5 ± 0.1	3.0 ± 0.2
*h*HDAC2	ND	>20	>20
*h*HDAC3	ND	4.8 ± 0.5	3.0 ± 0.1
*h*HDAC4	ND	>20	>20
*h*HDAC6	ND	>20	>20
*h*HDAC8	0.724 ± 0.0001	0.0057 ± 0.0001	0.027 ± 0.003
*h*HDAC10	ND	>20	>20
*h*HDAC11	ND	>20	>20

^1^ HeLa nuclear extract (rich in HDAC1 and HDAC2). ^2^ IC_50_ values are expressed as mean ± standard deviation of three independent experiments. ND: not determined.

#### 5.1.4. Linkerless HDAC8 Inhibitors

**Figure 7:**
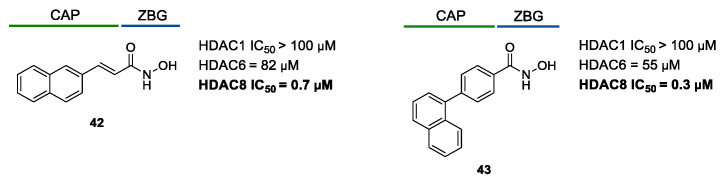
Structures and IC_50_ of **42** and **43** [150].

Among the available collection of the hydroxamic acid-based HDAC8is, linkerless inhibitors are worth mentioning since they do not fully comply with the canonical pharmacophore model of HDACis. In this specific case, the activity and selectivity towards HDAC8 exclusively rely on the ZBG and the cap group. These molecules represented valuable tools for the investigation concerning the structural and conformational aspects, offering insights into isoform-selective HDAC8 inhibition. In 2007, a six-membered class of compounds was rationally designed by combining the hydroxamate motif with different bulky hydrophobic caps. Screening by enzymatic assay identified **42** and **43** as potent (IC_50_ = 0.7 μM, IC_50_ = 0.3 μM, respectively) and >100-fold selective HADC8is over the isoforms 1 and 6 (Figure 7) [150]. 

The compounds contain a naphthyl group linked to the ZBG through a vinyl or a phenyl moiety: in silico studies demonstrated that the resulting structures are able to interact with an HDAC8 specific pocket. These data were corroborated by the X-ray crystal structure of compound **43** in complex with HDAC8 (PDB code: 5FCW) that have contributed to extend the knowledge of HDAC8 inhibition at the molecular level [22].

### 5.2. HDAC8is Bearing Novel ZBGs 

Many attempts were accomplished to find new valuable ZBGs endowed with chelating properties which can be comparable to hydroxamic acid. However, to date, few examples are reported in literature, and the inhibitory potency registered for the relative compounds remains in the micromolar range. In this frame, azetidine-2-one, amino acid residues, and thiazolidinedione were proposed as potential ZBG in HDAC8is. 

**Figure 8:**
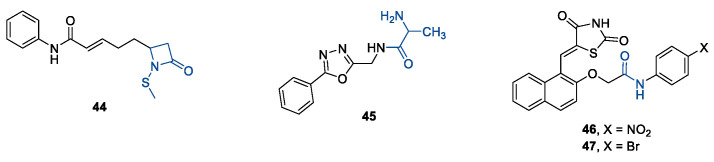
Structure of non-hydroxamate HDAC8 inhibitors (**44**–**47**); the predicted ZBGs are in blue [151,152,153,154,155].

Azetidin-2-one is a privileged ring in medicinal chemistry which was introduced as innovative ZBG in HDAC8is. Proper modification of the nitrogen with thiomethyl group led to the discovery of the HDAC8i **44** which exhibited a moderate inhibitory activity (IC_50_ 4.53 μM), and selectivity over the other HDACs (IC_50_ > 1000 μM) (Figure 8). Computational investigation has enlightened that the carbonyl of the azetidin-2-one is capable of chelating the zinc in a monodentate fashion, explaining the low potency of this inhibitor, while the sulphur atom is involved in the interaction with Trp141 of HDAC8. The compound displayed restrained cytotoxicity with an IC_50_ of 10–28 μM against SH-SY5Y neuroblastoma cell line [151]. 

Pidugu and co-workers designed oxadiazole-based derivatives conjugated with glycine or alanine. The rational design relied on the potential chelating properties of amino acid residues: to validate this hypothesis, molecular docking studies were performed, showing that the amide motif is involved in the coordination of the zinc in HDAC8 active site. Among the set of compounds, **45** exhibited the highest HDAC8 inhibitory potency (IC_50_ = 98 nM) and antiproliferative activity against MDA-MB-231 breast cancer cell line (IC_50_ = 230 nM) (Figure 8). However, further structural optimization is required to improve the selectivity of this class of derivatives over the other HDACs [152,153]. 

Recently, a class of novel 5-naphthylidene-2,4-thiazolidinedione was prepared and evaluated for their inhibitory activity against HDAC8. Although the thiazolidinedione motif was incorporated as ZBG, preliminary docking studies have enlightened that the carbonyl of the amide group is the moiety that coordinates the zinc ion in the active site in a monodentate fashion. Compounds **46** and **47** showed a moderate activity towards HDAC8 (IC_50_ values of 2.7 μM and 6.3 μM, respectively) coupled with an interesting selectivity over isoforms 1–6 (Figure 8) [154,155].

In 2016, benzothiazine was employed as a scaffold in a new class of potential HDAC8is. Despite that they are not structurally related to other known HDACis, some derivatives emerged for their capacity in selectively inhibiting HDAC8. In particular, the screening on an extended panel of HDACs (1–8) indicated **48** (Table 9) as the best performing compound, while **49** (Table 9) displayed the highest selectivity in Jukart cell line [156]. Further SAR investigation led to identification of the 3-benzo-thiazine-2-thione **50** (Table 9), emerging for its capability in reducing clonogenic growth of SK-N-BE(2)-C with an GI_50_ of 6 μM. The hypothesis that the thione sulphur could act as ZBG was computationally investigated by molecular docking [157].

## 6. Multi-Target Pharmacological Tools Acting on HDAC8

### 6.1. Multi-Drug Combinations Targeting HDAC8

While the development of selective and potent HDAC8is both as chemical probes and drug candidates remains crucial, the multi-target pharmacological approach on HDAC8 has recently gained attention since achieving the simultaneous modulation of multiple targets could offer several benefits, especially in complex diseases such as cancer, CNS diseases and fibrosis [158].

Combination therapy is a well-established pharmacological strategy in which different therapeutic agents are simultaneously administered to obtain additive or synergistic effects. Several studies showed the potential benefits of an HDAC8-targeting multi-drug therapy in different kinds of solid and liquid tumours. A combination of PCI-34051 (**1**), a selective HDAC8i, and cytarabine, the standard chemotherapeutic agent used in AML first-line therapy, was shown to have a synergistic effect in the reversion of myeloid leukaemia phenotype in AML cell line, promoting apoptosis in p53-expressing THP-1 cell line and cytostatic effect in HL60 cells [94]. A similar synergistic antiproliferative effect was highlighted in the HCC1806 breast cancer cell line after treatment with a combination of PCI-34051 (**1**) and the conventional cytotoxic multi-drug cocktail composed of cyclophosphamide, adriamycin, and 5-fluorouracil (CAF), while the single administration of the HDAC8i yielded no significant effect [93]. HDAC8-targeting combined treatment was also investigated in neuroblastoma models: BE(2)-C neuroblastoma xenografts were simultaneously treated with PCI-48012 (the structure of which is still undisclosed), a more stable analogue of PCI-34051 (**1**), and retinoic acid. A significant synergistic reduction in the tumour volume was demonstrated, especially when compared to the ones treated exclusively with retinoic acid [111]. In 2018, Shen et al. demonstrated that PCI-34051 (**1**), in combination with the anaplastic lymphoma kinase/tyrosine-protein kinase Met (ALK/MET) inhibitor crizotinib, efficiently inhibited the activation of growth receptor survival signalling, leading to cell cycle arrest in neuroblastoma cell lines and reducing tumour cell growth in a zebrafish xenograft model [112]. These findings provide a solid rationale for the future development of novel synergistic drug combinations targeting HDAC8 for the treatment of different pathological conditions. 

### 6.2. Polypharmacological Tools Targeting HDAC8

**Figure 9:**
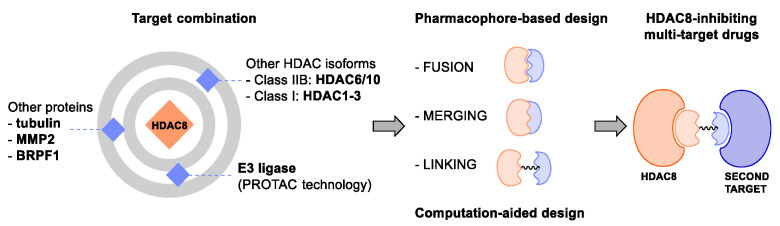
Schematic representation of the biological targets recruited by the reported polypharmacological tools alongside HDAC8, the suitable approaches to their design and their mode of action.

Polypharmacological tools engaging HDAC8 have also been developed since multi-target chemical entities represent an attractive and promising approach for the treatment of multifactorial diseases [159]. This innovative multi-target approach could overcome some of the inconveniences of multi-drug therapies, including drug resistance onset, high dosages, additive toxicities, undesired side effects and drug–drug interactions while improving treatment efficacy and patient compliance [158]. A brief overview of the HDAC8-inhibiting multi-target drugs reported in the later sections is outlined in Figure 9, with a schematic representation of the feasible design strategies and mode of action.

#### 6.2.1. Selective HDAC6/8 Dual Inhibitors

Given the highly conserved nature of the catalytic deacetylase domain among all HDAC isoforms, the challenge of isoform-selectivity has been overturned and exploited to develop inhibitors able to modulate different isozymes, to achieve improved therapeutical efficacy while avoiding the typical side effects of the so-called promiscuous drugs. As evidence highlighted that simultaneous inhibition of HDAC6 and HDAC8 could lead to synergistic therapeutic effects in cancer and could be beneficial in different pathologic conditions, several dual HDAC6/8 inhibitors have been recently disclosed [7,160,161].

**Figure 10:**
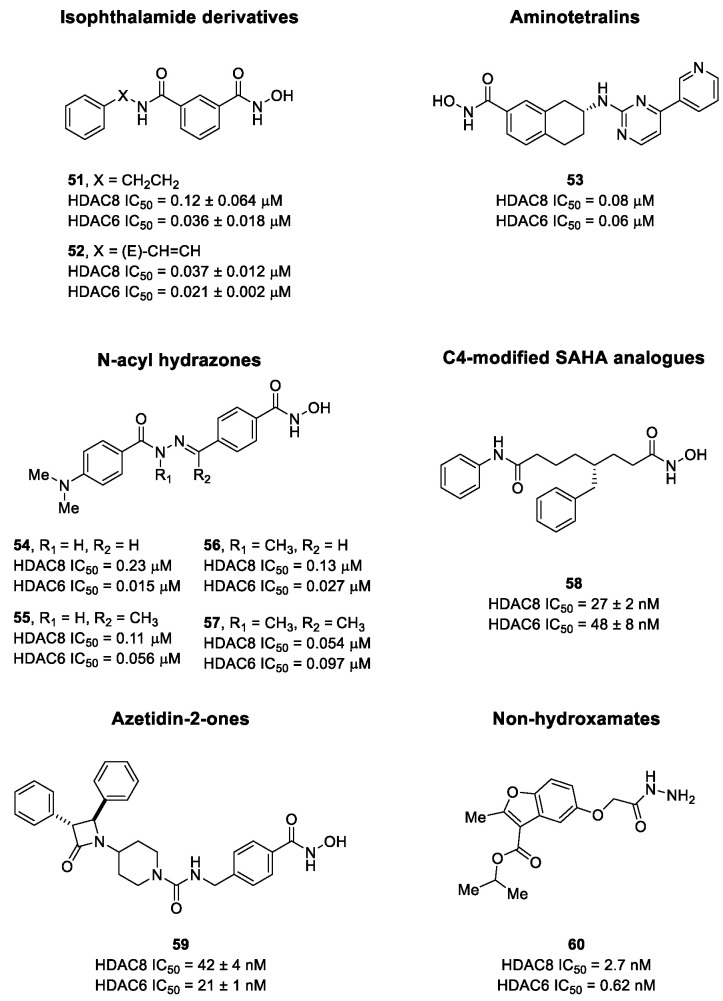
Representation of reported dual HDAC6/8is [21,36,160,161,164,169].

Olson et al. discovered the first dual HDAC6/8 inhibitor, BRD-73954 (**51**, Figure 10), while exploring positional modifications on an in-house selective HDAC6 inhibitor previously identified [162]. The transposition of the hydroxamic acid moiety from the para to the meta position of the phenyl ring resulted in an increased inhibitory activity on HDAC8 (IC_50_ = 0.12 ± 0.064 μM) while retaining potency on HDAC6 (IC_50_ = 0.036 ± 0.018 μM), with good selectivity over HDAC2/4. The rigid derivative **52** (Figure 10) showed increased inhibitory activity on both target isoforms with an IC_50_ on HDAC6 of 0.021 ± 0.002 μM and HDAC8 of 0.037 ± 0.012 μM. Biochemical HeLa cell assays confirmed the engagement of the two enzymes [160].

In 2014, Olson et al. developed an aminotetralin-based library of HDAC6/8 selective inhibitors by scaffold hopping starting from a focused library of tetrahydroisoquinolines, which showed high tubulin acetylation and low p21-induction in cellular assays. The R-enantiomer **53** (Figure 10) proved to be the most potent compound of the series, selectively inhibiting HDAC6 and HDAC8 with IC_50_ values of 0.05 μM and 0.08 μM, respectively, when tested against HDAC1-11. Computational docking of both enantiomers demonstrated that the HDAC8 catalytic pocket demanded stricter structural requirements compared to HDAC6, as the two stereoisomers only took optimal binding poses in the HDAC6 homology model [36,163].

Structural modification of the selective class I/II HDAC inhibitor TSA led to the discovery of a novel class of dual HDAC6/8 inhibitors, consisting of *N*-acylhydrazone derivatives of the parent drug. Compounds bearing the hydroxamic acid moiety in *meta* and *para* position of the phenyl ring were designed, and biological tests on *h*HDAC isoforms highlighted that *para*-substituted derivatives were more potent that their *meta* regioisomers. Single and double methylation of the hit compound **54** (Figure 10, HDAC6 IC_50_ = 0.015 μM; HDAC8 IC_50_ = 0.23 μM) on the hydrazonic C and N was then examined and resulted in compounds **55** (Figure 10, HDAC6 IC_50_ = 0.056 μM; HDAC8 IC_50_ = 0.11 μM), **56** (Figure 10, HDAC6 IC_50_ = 0.027 μM; HDAC8 IC_50_ = 0.13 μM) and **57** (Figure 10, HDAC6 IC_50_ = 0.097 μM; HDAC8 IC_50_ = 0.054 μM). Compound **57** emerged as a more balanced dual inhibitor, maintaining a good selectivity profile against HDAC1/2. The *N*-methylated analogues showed a different lowest-energy conformation of the C=N double bond, switching from *s-cis* to *s-trans* to avoid steric hindrance caused by the proximity of the methyl and phenyl groups. Antiproliferative activity of compounds **55**–**57** was also evaluated through MTS assay on HepG2 and HT144 hepatocellular carcinoma cell lines, highlighting how these derivatives could selectively interrupt the proliferation of certain hepatocellular carcinoma cells [164].

In addition to TSA, SAHA proved to be a suitable scaffold to perform structural modifications leading to dual HDAC6/8 inhibition. To analyse the essential structural requirements to achieve selectivity in HDAC inhibition, several substitutions of C2-C6 in the linker region were explored by Pflum and co-workers in the last fifteen years [35,165,166,167,168]. C4-modified SAHA analogues turned out to be the most potent and selective suberoylanilide derivatives. These compounds showed nanomolar inhibitory potency towards HDAC6 and HDAC8, compared to HDAC1-3, during HDAC inhibition tests. Among the proposed substitutions, which include ethyl, *n*-butyl, *n*-hexyl, phenyl, and benzyl moieties, the R-enantiomer of benzyl analogue **58** (Figure 10) was the best-performing derivative, inhibiting HDAC6 and HDAC8 with an IC_50_ value of 48 ± 8 and 27 ± 2 nM, respectively, and exhibiting 1300-fold selectivity against HDAC1-3. The S-distomer of **58** and C4-*n*-butyl analogue still showed nanomolar dual HDAC6/8 inhibition but slightly lower potency and selectivity. Racemic C4-benzyl SAHA analogue was also tested in U937 leukaemia cells for HDAC isoform-selectivity and cell growth inhibition, marking these compounds as useful chemical tools and potential anti-cancer pharmacological agents [161].

Recently, a library of diphenyl-azetidin-2-one-based dual HDAC6/8 inhibitors was disclosed as potential anticancer agents. Among the designed compounds, derivative **59** (Figure 10), bearing two *trans* phenyl rings at positions C3 and C4 of the azetidine-2-one core and a urea moiety tethering the bulky cap and benzyl linker, proved to be the most promising of the series. It exhibited an IC_50_ of 21 ± 1 nM on HDAC6 and of 42 ± 4 nM on HDAC8, with a 100-fold selectivity over HDAC1 (IC_50_ = 4200 nM). Docking studies revealed the binding mode of the compound and the increased acetylation levels of α-tubulin and SMC3 by Western blot analysis confirmed dual HDAC6/8 inhibition. Compound **59** was then tested on U937 and HCT116 cells, proving its antiproliferative therapeutic effects on both haematological and solid cancers. In addition, solubility, chemical and metabolic stability of **59** were also assessed. Evaluation of toxicity in mouse fibroblasts and zebrafish embryos confirmed the tolerability of this compound in normal cells [21].

Despite the high potency of inhibition ensured by the hydroxamic acid ZBG, continuous endeavours are made to discover different ZBGs that could overcome the genotoxicity and pharmacokinetic issues that are linked to the widely employed hydroxamic moiety.

During the development of selective HDAC8is by in silico screening and in vitro biological evaluation, compound **60** (Figure 10, HDAC6 IC_50_ = 0.62 nM; HDAC8 IC_50_ = 2.7 nM) emerged as a potent non-hydroxamic HDAC6/8 dual inhibitor. This compound was discovered by computational studies that initially focused on the selection of a predictive pharmacophore hypothesis for selective HDAC8 inhibition, starting from the structurally diverse 32 known selective HDAC8is. The most predictive hypothesis was then used to build a 3D QSAR model. Subsequently, virtual screening of the Phase database, in silico ADMET optimization of the fitting hits, and docking studies with HDAC8 and other HDAC isoforms followed, yielding five non-hydroxamate hit compounds, which were then synthesized and exclusively tested in vitro on HDAC isoforms 6 and 8 [169].

#### 6.2.2. Selective HDAC6/8/10 Multi-Target Inhibitors

**Figure 11:**
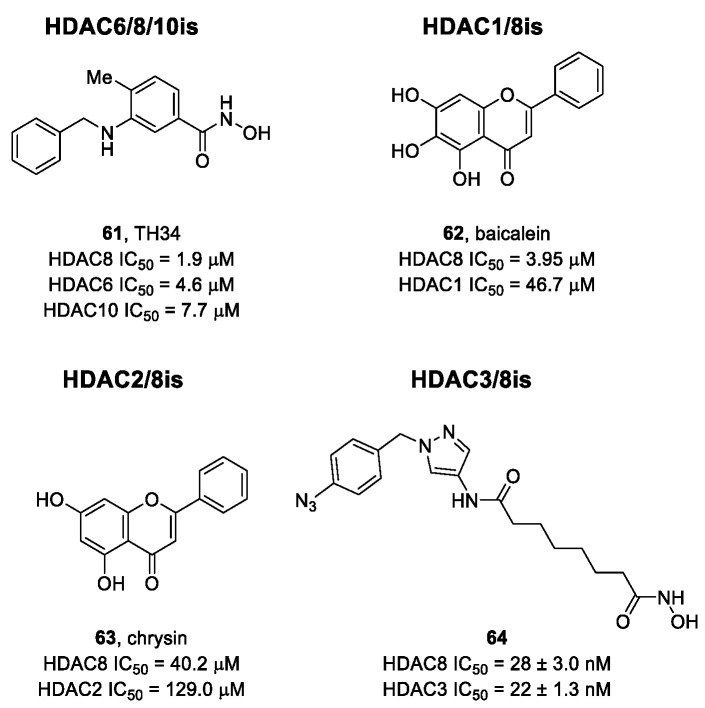
Representation of reported multi-target HDAC6/8/10is and dual HDAC1-3/8is [170,175,176,178].

In 2018, Kolbinger et al. disclosed the structure of TH34 (**61**, Figure 11), a novel HDAC6/8/10 inhibitor capable of selectively inhibiting the three isoforms in low-micromolar concentrations (HDAC6 IC_50_ = 4.6 μM; HDAC8 IC_50_ = 1.9 μM; HDAC10 IC_50_ = 7.7 μM), as a potential therapeutic agent for the treatment of high-grade neuroblastoma. It was shown that a combination of TH34 and all-*trans* retinoic acid synergistically inhibited colony growth of neuroblastoma cell lines.

Evidence highlighted that HDAC8 is overexpressed in high-grade neuroblastoma and that treatment with PCI-34051 (**1**) induced cellular differentiation. Furthermore, HDAC8/10 expression is strongly correlated with markers of poor prognosis and overall survival; thus, a dual HDAC8/10 could be beneficial in neuroblastoma therapy. Due to the similarity of HDAC6 and 10 highly conserved catalytic domains, segregating inhibitory activity on the two isoforms was found to be challenging. However, given that HDAC6 is not significantly correlated to prognostic markers in neuroblastoma and its inhibition is well tolerated in preclinical and clinical studies, even a triple-acting inhibitor could exert the same favourable impact, without many side effects. Neuroblastoma cell lines treated with TH34 showed signs of neuronal differentiation, by inducing DNA damage and caspase-dependent cell death, as confirmed after treatment and co-incubation of cells with TH34 and Z-VAD-FMK, a pan-caspase inhibitor. Moreover, TH34 showed limited cytotoxic effects on proliferating non-malignant fibroblasts [170].

#### 6.2.3. HDAC1-3/8 Dual Inhibitors

Baicalein (**62,** Figure 11), a natural trihydroxyflavone extracted from *Scutellaria baicalensis* and *Scutellaria lateriflora* has been under investigation for the last ten years due to its antineoplastic, antiviral, anti-inflammatory, and antioxidant proprieties [171,172,173,174]. Recently, it emerged that baicalein also acts as a non-hydroxamic dual HDAC1/8 inhibitor and has anti-leukemic effects on AML cell lines, with an IC_50_ of 46.7 μM and 3.95 μM, respectively and good selectivity over HDAC2. [175]. A structurally related flavonoid extracted from propolis, chrysin (**63,** Figure 11), was formerly investigated as a potential HDAC inhibitor [176,177]. Chrysin was proven to be an HDAC2/8 dual inhibitor (HDAC2 EC_50_ = 129.0 μM; HDAC8 EC_50_ = 40.2 μM), without showing any activity on HDAC1, and was able to suppress cell growth while promoting differentiation in human breast cancer MDA-MB-231 cells. Moreover, oral administration of chrysin (90 mg/kg/day) to MDA-MB-231 xenograft mice led to significant inhibition of tumour growth [177]. Given that baicalein and chrysin only differ in the presence of the 6-hydroxy group, it can be deduced that this moiety is accountable for the shift in isoform selectivity between HDAC1 and 2, while retaining activity on HDAC8. Taking into consideration the privileged nature of this versatile flavone scaffold, future developments of baicalein- and chrysin- derived multi-target inhibitors acting on HDAC8 cannot be excluded.

A library of HDAC3/8 dual-acting inhibitors was designed by Neelarapu et al. as pyrazole-based azide probes, exhibiting low nanomolar activity on both isoforms. These compounds are suitable for binding ensemble profiling with photoaffinity labelling (BEProFL) experiments in cells and showed antiproliferative and neuroprotective effects at a micromolar concentration by inhibiting nuclear HDACs, as confirmed by tests on HepG2 cells. Compound **64** (Figure 11) presented an IC_50_ of 22 ± 1.3 nM on HDAC3 and of 28 ± 3.0 nM on HDAC8, with an acceptable selectivity over HDAC1, 2, 4–7, 9–11. Docking studies highlighted that pyrazole-based azide compounds, unlike their isoxazole analogues, can occupy a second binding pocket in HDAC8 increasing the number of positive interactions, owing to their flexibility [178].

#### 6.2.4. Tubulin Polymerization and HDAC8 Dual Inhibitors (TP/HDAC8is)

**Figure 12:**
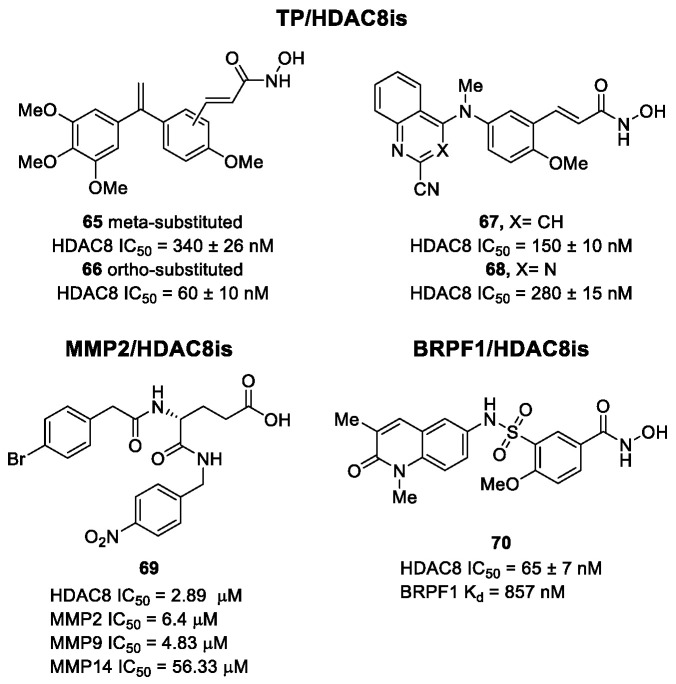
Representation of reported dual TP/HDAC8is, MMP2/HDAC8is and BRPF1/HDAC8is [179,180,184,185].

Chimeric molecules capable of inhibiting both tubulin polymerization and HDAC8 activity have been developed in 2018 from semisynthetic *iso*-combretastatin A-4 (*iso*CA-4), through a pharmacophore merging approach in which hybrid compounds were designed by linking *iso*CA-4 to a hydroxamic acid ZBG. Compounds **65** and **66** (Figure 12) displayed high potency of inhibition on HDAC8 (IC_50_ = 340 ± 26 and 60 ± 10 nM, respectively) compared to HDAC6/11 and were tested on various human tumour cell lines, confirming their antiproliferative activity, which resulted from the disruption of microtubule organization and the cell cycle arrest of tumour cells at the G2/M phase. Compound **65** was able to exert a good antiproliferative effect on HT-29 cells, belonging to a line of combretastatin A-4 refractory human colon adenocarcinoma cells. It emerged as 135-fold more active than *iso*CA-4 and 17-fold than TSA and was proved to be as potent as a combination of *iso*CA-4 and TSA, confirming the therapeutic potential of this compound [179].

Recently, a novel series of quinoline-2-carbonitrile-based hydroxamic acids was also disclosed as TP/HDAC8is. Starting from *iso*CA-4 and the first generation of dual TP/HDAC8is, the compounds were designed by substituting the 3,4,5-trimethoxyphenyl A-ring with a quinazoline or quinoline ring. Compounds **67** and **68** (Figure 12) emerged as the most promising derivatives, showing the best cytotoxic activity against a panel of human cancer cell lines, which included both liquid (leukemia) and solid (stomach, pancreas, ovarian, lung, breast, colon) tumours. In these cellular assays, both compounds showed sub-nanomolar averaged IC_50_ (0.6 and 0.7 nM, respectively). The dual mode of action was confirmed by evaluating both G_2_/M cell cycle arrest and apoptosis caused by microtubule disruption, on HT-29 cells, and HDAC inhibition in a global in vitro inhibitory activity assay. Compounds **67** and **68** were found to be selective HDAC8is, with IC_50_ of 150 ± 10 and 280 ± 15 nM, respectively. Compound **68** proved to be more selective against HDAC6 (IC_50_ = 2000 ± 45 nM) and HDAC11 (IC_50_ = 3200 ± 100 nM), while compound **67** only showed about seven-fold HDAC8 selectivity over HDAC6 and 13-fold selectivity over HDAC11. The anti-proliferative and anti-tubulin activities positively correlated with the effects observed on cell-cycle progression and HDAC8 inhibition. In addition, metabolic studies proved the enhanced stability of this second-generation dual TP/HDAC8is [180].

#### 6.2.5. MMP2/HDAC8 Dual Inhibitors

Matrix metalloproteinases are zinc-dependent endopeptidases involved in the degradation of extracellular matrix and remodelling of tissues. MMP2, also called gelatinase A, is strongly correlated with cancer progression, as it is rapidly produced by growing tumour cells. Being one of the main molecular targets of cancer metastasis, attempts to design potential MMP2 inhibitors have been made in the last few decades [181]. All these potential pharmacological agents have failed in clinical trials due to their broad spectrum of inhibition, leading to undesirable adverse effects. Since both HDACs and MMPs are metalloenzymes that play a role in cancer migration, invasion, angiogenesis, and metastasis, dual MMP2/HDAC8is were designed to enhance the anti-migratory, and anti-invasive properties of selective MMP or HDAC inhibitors. Moreover, HDAC8, along with isoforms 1 and 6, was proven to increase invasion and MMP9 expression in breast cancer cells, and together with MMP2, is overexpressed in AML and acute lymphoblastic leukaemia (ALL) [182,183].

Ligand-based pharmacophore mapping and molecular docking were employed to design molecules capable of retaining the structural requirements needed for MMP2 inhibition while satisfying the HDAC8i pharmacophoric demands, starting from antineoplaston metabolite, N2-phenylacetyl-L-*iso*-glutamine. Tailoring of the selective hits led to a focused library of 35 potential dual-acting compounds. Derivative **69** (Figure 12) emerged as the most promising, with an IC_50_ of 2.89 μM against HDAC8, no activity against nuclear HDACs, and non-selective inhibitory activity on MMP2/9/14 (IC_50_ MMP2 = 6.4 μM; IC_50_ MMP9 = 4.83 μM; IC_50_ MMP14 = 56.33 μM) and weak inhibition of MMP8/12 (IC_50_ MMP8 = 205.89 μM; IC_50_ MMP12 = 138.32 μM). Compounds simultaneously inhibiting MMP2/9 and HDAC8 were more potent than their MMP2/9 or HDAC8 selective counterparts. Compound **69** was then tested for its anti-proliferative, anti-migratory and anti-invasive properties on lung carcinoma A549 cells. Results of the MTT test, which showed negligible cytotoxicity at 50–100 μM, the wound healing assay, which denoted a prominent anti-migratory effect, and the QCM ECMatrix cell invasion assay, which showed 38–42% of reduction in cell invasion, confirmed the aforementioned features and paved the way for the discovery of novel dual MMP2/HDAC8is [184].

#### 6.2.6. Bromodomain BRPF1 and HDAC8 Dual Inhibitors

Bromodomains (BRDs) are reader proteins that, along with writer (histone acetyltransferases, HATs) and eraser proteins (HDACs), control the state of histone acetylation, which is one of the most studied pathways of epigenetic control of gene expression. BRDs are usually an integral part of large protein complexes, and they specifically recognize ε-*N*-acetylated lysine residues, promoting the recruitment of transcription factors. Bromodomain and PHD finger-containing proteins (BRPFs) are a family of epigenetic proteins with multiple reader domains, including a bromodomain, acting as scaffolds for the recruitment and assembly of HATs belonging to the MYST family. As HATs activity is dysregulated in cancer and causes overexpression of oncogenes in different tumours, especially leukaemia, BRPF1 has been exploited as a biological target for the development of potent and selective BRPF and BRPF1 inhibitors. Evidence shows that combination therapies targeting different epigenetic modulators could result in better clinical results than monotherapy. Thus, in 2020, Ghazy et al. exploited the polypharmacological approach to design dual HDAC/BRD inhibitors. The benzhydroxamic scaffold of selective HDAC8is was used to perform structural modifications arising from the inclusion of a BRPF1 inhibiting 1,3-dimethylquinolin-2-one moiety. Molecular modelling yielded chimeric compounds that merged HDAC8 and BRPF1 inhibiting structural features. Among the synthesized derivatives tested in enzymatic assays, sulfonamide compound **70** (Figure 12) emerged as the most potent compound on HDAC8 (IC_50_ = 65 ± 7 nM), with a K_d_ on BRPF1 of 857 nM, while maintaining over 10-fold selectivity against HDAC1/6 [185].

#### 6.2.7. Selective HDAC8-Degrading PROTACs

Proteolysis targeting chimeras (PROTACs) represent an innovative approach to epigenetic modulation, and owing to their structure, they can be considered dual-acting compounds. They are small hetero-bifunctional molecules consisting of three structural elements: a ligand capable of recruiting ubiquitin ligase E3, a ligand that binds to the protein of interest (POI), and a suitable linker that tethers the first two elements. PROTAC technology is based on a reversible chemical knockdown strategy, in which a specific protein that is involved in the pathogenesis or the exacerbation of a disease is degraded by the ubiquitin–proteasome system, after the formation of a bioactive ternary complex. The PROTAC catalytic mechanism of action is based on the simultaneous recruitment of the POI and the E3 ligase, which “flags” the target protein promoting its ubiquitination and subsequent degradation by the proteasome. This cutting-edge technology presents some significant benefits compared to small molecules: prevention of drug resistance development, suppression of both catalytic and non-catalytic functions of the target protein, and ability to target “undruggable” target proteins with enhanced selectivity [186].

**Figure 13:**
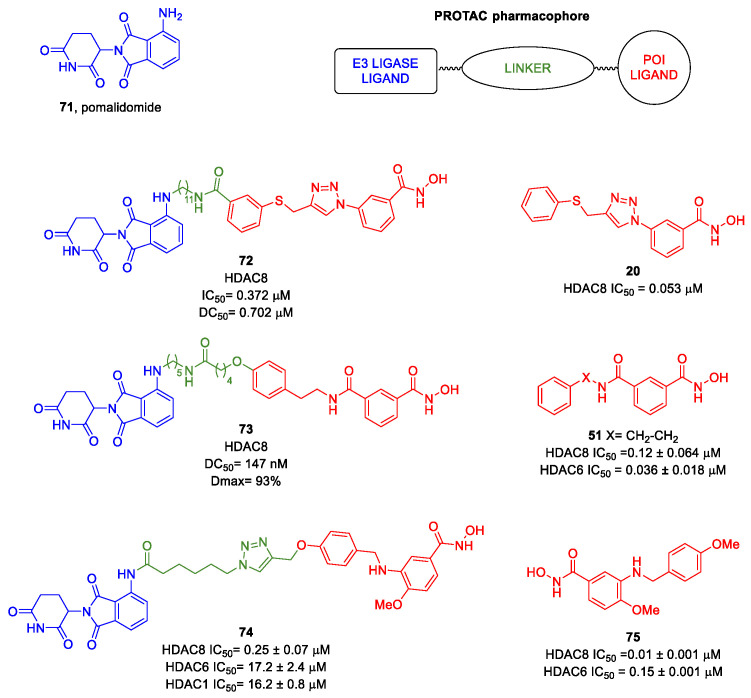
Representation of reported HDAC8-degraders and HDAC8-inhibiting precursors [142,143,160,187,188,189].

Recently, the first HDAC8 selective PROTACs have been disclosed. The design of these novel degraders started from docking studies of the previously discovered benzhydroxamic selective HDAC8i **20** (Figure 13, HDAC8 IC_50_ = 0.053 μM) [142]. The *meta*-position of the terminal phenyl ring of **20** was chosen as an anchoring point to attach the linker, since the other positions appeared more hindered in docking studies. Three alkyl linkers (C5, C8, C11) were selected to evaluate how the linker length affected the ternary complex formation and subsequent potency of degradation. Pomalidomide (**71**, Figure 13) was chosen as the cereblon (CRBN) E3 ligase ligand. Three compounds were synthesized and tested on T-cell leukaemia Jurkat cells, to assess the HDAC8 degradation. PROTAC **72 (**Figure 13), with a C11 linker, showed the most potent HDAC8 degrading activity (IC_50_ = 0.372 μM; DC_50_ = 0.702 μM). The ubiquitin–proteasome system (UPS)-dependent PROTAC mechanism of action was confirmed by Western blot, co-treating Jurkat cells with MLN7243, a ubiquitin-activating enzyme inhibitor, or bortezomib, a proteasome inhibitor, and observing a reduction of HDAC8 degradation, induced by 10 μM concentration of the compound. Inhibition of Jurkat cancer cells growth was also assessed, and PROTAC **72** proved to be 10 times more potent than the parent compound **20**, showing a GI_50_ of 7.09 μM. Moreover, compound **72** did not influence the levels of HDAC1/2/6. A *para*-substituted analogue, which proved to be inactive, was also synthesized to confirm that the meta position was the most favourable to obtain HDAC8 degradation. [187].

A library of HDAC8-degrading PROTACs has been developed by Sun et al. using BRD73954 (**51**), a dual HDAC6/8 inhibitor, as warhead and pomalidomide as the E3 ligase ligand. The linker moiety was installed on the *para*-position of the terminal phenyl ring through an ether bond, and preliminary screening of the linker length was performed, yielding more than 20 compounds bearing a flexible linker, varying in length. Compounds with a more rigid structure tethering pomalidomide and the linker chain were also synthesized, as well as a 5-substituted pomalidomide-based PROTAC and two negative controls bearing a masked hydroxamic acid. Among the designed flexible compounds, PROTAC **73** (Figure 13; DC_50_ = 147 nM; D_max_ = 93%) was the most effective HDAC8 degrader, showing 85.0 ± 0.4% of HDAC8 degradation in HCT-116 cells at 5 μM. The rigid derivatives were also tested on HCT-116 cells to evaluate HDAC8 degradation and compound **73** proved to be the best-performing of the series, producing 74.4 ± 0.8% of HDAC8 degradation at 5 μM. Compound **73** was then used to investigate the time-course change of HDAC8 protein expression in the same cell line, and this Western blot assay showed that the maximal degradation effect was achieved 10 h after treatment. A quantitative analysis of HDAC8 levels in HCT-116 cells 10 h after treatment with different concentrations of **73** was also carried out. PROTAC **73** was then tested for selectivity against different HDAC isoforms, and Western blot analysis confirmed that HDAC1/3 expression was not affected at all tested concentrations. Even if the HDAC6/8 dual inhibitor BRD73954 is more potent on HDAC6 than on HDAC8, it was proven that HDAC6 degradation was 30-fold weaker than that of HDAC8, with the latter happening at higher concentrations. The negative controls were not able to degrade HDAC8, as expected and co-treatment with **73** and BRD73954 (**51**) or pomalidomide suppressed HDAC8 degradation, since simultaneous recruitment of the E3 ligase and POI is necessary. The mechanism of action was also confirmed by co-treating HCT-116 cells with PROTAC **73** together with MG132, a proteasome inhibitor, or bafilomycin A1, a lysosome inhibitor. Only co-treatment with MG132 led to suppression of HDAC8 degradation, suggesting a UPS-mediated target degradation [188].

Investigation of the potential anti-neuroblastoma activity of newly designed HDAC8 degraders was pursued by Darwish et al. [189], due to the critical involvement of HDAC8 in this pathology. The developed PROTACs were designed starting from previously disclosed benzhydroxamic HDAC8 inhibitors [143,189]. Different E3 ligase ligands, including CRBN and VHL, were explored to evaluate how the target degradation would be affected. Application of hydrophobic tagging (HyT) PROTAC technology, in which proteasome-dependent degradation is induced by inducing or mimicking protein misfolding, yielded an additional four-membered class of derivatives. The best-performing compounds of the series were the CRBN-recruiting ones, and PROTAC **74** (Figure 13) showed an IC_50_ of 0.25 ± 0.07 µM against HDAC8, while maintaining good selectivity against HDAC6 (IC_50_ = 17.2 ± 2.4 µM) and HDAC1 (IC_50_ = 16.2 ± 0.8 µM). Compound **74** was designed by tethering pomalidomide (**71**) to the para-position of the phenyl cap group of compound **75** (Figure 13), through a triazole-based alkyl linker. This position was chosen as an anchoring point given the exposure to the exit of the binding tunnel highlighted in molecular docking studies. In addition to in vitro screening, cellular testing on neuroblastoma SK-N-BE(2)-C cells was carried out to assess the effect of the developed PROTACs on colonogenic growth of tumour cells. Compound **74** showed a strong effect on colony formation, indicating effectiveness in the impairment of cell survival and proliferation, but exhibited weak to no cytotoxic effects against HEK293 cells at 50 µM concentration. Treated whole SK-N-BE(2)-C cell lysate was then used to evaluate HDAC8 degradation, and after 10 h of treatment at 10 µM, HDAC8 levels were reduced down to 30%. PROTAC **74** also showed strong hyperacetylation of SMC3, as opposed to the negative control bearing an ester in place of the hydroxamic acid motif. Treatment of neuroblastoma cells with PROTAC **74** also led to neuronal differentiation, as confirmed by neurite-like outgrowths and enhanced differentiated phenotype in microscopic analysis [189]. These results confirm the potential therapeutic application of HDAC8-degrading PROTACs in neuroblastoma and pave the way towards the future development of novel epigenetic degraders for the treatment of complex diseases.

## 7. Conclusions

HDAC8 is involved in different pathways underlying the onset and progression of different diseases, and in recent years, it emerged as a valuable target in medicinal chemistry. To date, HDAC8 activation/inhibition represents a potential tool to counteract X-linked disorders, aberrant wound healing, cancer, and neurological disorders. Concerning these latter, further studies are required to clarify the role of HDAC8 in neuropathological conditions and the mechanism by which its inhibition might exert a therapeutic effect. In general, all the observations reported in this overview confirmed the versatility of selective HDAC8is as valuable pharmacological tools for increasing therapeutic interventions, encouraging the research to unveil the molecular bases underlying HDAC8 inhibition.

A mounting body of evidence highlighted the benefits which could derive from isotype-selective targeting; thus, drug design and development of novel HDAC8 inhibitors focused on the achievement of an acceptable activity/selectivity profile in vitro and in vivo. To this purpose, many endeavours were accomplished to identify the structural differences among the HDAC isoforms which might be exploited to attain a selective inhibition. In this regard, crystallographic studies, assisted by computational tools, allowed for identifying a specific HDAC8 sub-pocket that could be exploited to obtain the challenging selectivity over the class I HDACs and HDAC6. Furthermore, the structure-based rational drug design can rely on the availability of known selective HDAC8is which enabled the drawing of general SARs. Although the current selective HDAC8is mostly adheres to the canonical 3-motif pharmacophore model (cap group, liker, ZBG), small structural and conformational modifications are crucial for the achievement of the desired selectivity. Among them, it was noted that compounds containing aromatic-based linkers combined with hydroxamic acid as ZBG exhibited higher inhibitory potency against HDAC8. Moreover, the *meta*-substitution of the ZBG, favouring the L-shaped conformation, ensures the discrimination among the other isozymes, especially HDAC6, which prefers a *para*-substitution.

The advances in drug design of selective HDAC8is have been paralleled to as many achievements in the biological field which are pivotal for proving the selective engagement of HDAC8 at the cellular level. In a drug discovery trajectory, the hit selection, based on specific enzymatic studies, should be followed by a hit validation that is currently executed by assessing the level of acetylation of the preferential substrates of HDAC8 namely SMC3 and H3. However, to exclude off-targets phenomena and to eventually attribute the observed biological effects to the exclusive inhibition of HDAC8, further biological options are highly desired for implementing the current armamentarium of reliable and exploitable cellular tests.

Recently, a multi-drug approach was pursued to develop novel synergistic drug combinations encompassing HDAC8 inhibition for the treatment of different pathological conditions. In this context, the early polyfunctional modulators, including PROTACs, showed to be effective in countering aberrant conditions associated to some diseases; in a perspective view, these approaches could represent a further implemented application of HDAC8 inhibition.

## Figures and Tables

**Table 1:** General structure and IC_50_ values of **14**, **15**, and **16** [139].

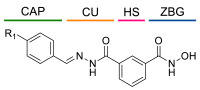				
Cpds	R_1_	*h*HDAC2 IC_50_ (μM)	*h*HDAC3/NCoR1 IC_50_ (μM)	*h*HDAC8 IC_50_ (μM)
**14**	H-	20	18	0.052
**15**	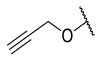	6.3	6.2	0.029
**16**	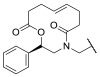	3.6	15	0.023

**Table 2:** General structure and IC_50_ values of HDAC8 inhibitors bearing five-membered-based linker (**7**, **13**, **17**–**20**) [141,142].

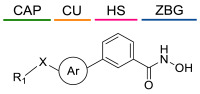								
Cpds	R_1_	X	Ar	*h*HDAC1 IC_50_ (μM)	*h*HDAC2 IC_50_ (μM)	*h*HDAC4 IC_50_ (μM)	*h*HDAC6 IC_50_ (μM)	*h*HDAC8 IC_50_ (μM)
**17**	Ph	-CH_2_	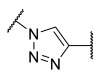	41	65	30	7.9	0.35
**18**	Ph	-CH_2_CH_2_	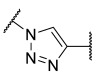	>100	76	>100	3.2	0.18
**13**	Ph	-SCH_2_	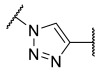	38	>100	44	2.4	0.070
**19**	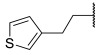	-CH_2_CH_2_	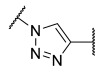	>100	>100	>100	1.1	0.10
**20**	Ph	-SCH_2_	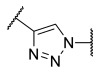	>100	>100	>100	2.2	0.053
**7**	Ph	-SCH_2_	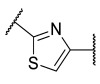	>100	>100	>100	14	0.15

**Table 3:** General structure and IC_50_ values of **21**, **22**, and **23** [143].

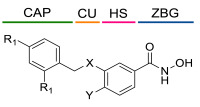						
Cpds	R_1_	X	Y	*h*HDAC1 IC_50_ (μM) ^1^	*h*HDAC6 IC_50_ (μM) ^1^	*h*HDAC8 IC_50_ (μM) ^1^
21	Cl	-NH	Me	18.7 ± 2.5	14.4 ± 2.4	0.035 ± 0.04
22	H	-NH	-OMe	14.5 ± 1.4	5.1 ± 0.8	0.069 ± 0.017
23	H	-O	-OMe	12.1 ± 5.7	2.9 ± 0.3	0.027 ± 0.03

^1^ IC_50_ values are expressed as mean ± standard deviation of at least two independent experiments.

**Table 4:** General structure and IC_50_ values of **24**, **25**, and **26** [34].

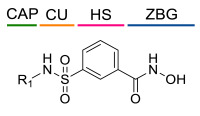				
Cpds	R_1_	*h*HDAC2 IC_50_ (μM) ^1^	*h*HDAC6 IC_50_ (μM) ^1^	*h*HDAC8 IC_50_ (μM) ^1^
**24**	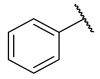	14.5 ± 2.4	1.5 ± 0.3	0.050 ± 0.010
**25**	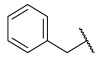	47.1 ± 7.0	2.6 ± 0.5	0.080 ± 0.020
**26**	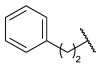	11.2 ± 1.8	1.8 ± 0.3	0.060 ± 0.010

^1^ IC_50_ values are expressed as mean ± standard deviation of at least two independent experiments.

**Table 5:** General structure and IC_50_ values of **27**, **28**, **29**, **30** and **31** [24].

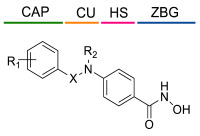							
Cpds	R_1_	R_2_	X	*h*HDAC3 IC_50_ (μM)	*h*HDAC6 IC_50_ (μM)	*h*HDAC8 IC_50_ (μM)	*h*HDAC11 IC_50_ (μM)
**27**	3-*t*Bu	H	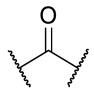	>1	0.066	0.0337	>1
**28**	3-*t*Bu	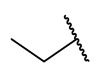	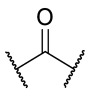	>1	>1	0.0835	>1
**29**	3-*t*Bu	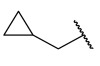	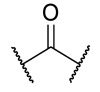	>1	>1	0.0660	>1
**30**	3,5-CF_3_	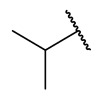	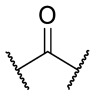	>1	>1	0.0234	>1
**31**	3-OCF_3_	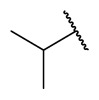	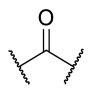	>1	>1	0.0655	>1

**Table 6:** General structure and IC_50_ values of **34**, **35**, **36** [145].

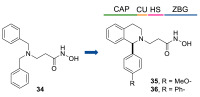					
Cpds	*h*HDAC1 IC_50_ (μM) ^1^	*h*HDAC2 IC_50_ (μM) ^1^	*h*HDAC3 IC_50_ (μM) ^1^	*h*HDAC6 (% Inhibition) ^2^	*h*HDAC8 IC_50_ (μM) ^1^
34	33 ± 1.1	2.7 ± 0.67	52 ± 3.0	97 ± 0.050	1.4 ± 0.41
35	27 ± 3.7	>100	>100	21 ± 0.040	0.082 ± 0.019
36	7.3 ± 0.48	47 ± 17	38 ± 2.2	39 ± 1.2	0.055 ± 0.014

^1^ IC_50_ values and ^2^ percent inhibition of HDAC6 activity at 10 μM concentration are expressed as mean ± standard deviation of at least two independent experiments.

**Table 7:** General structure and IC_50_ values of **37**, **38**, and **39** [146].

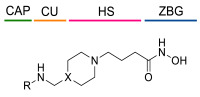					
Cpds	R	X	HeLa HDACs IC_50_ (μM) ^1^	*h*HDAC3/NCoR1 (% Inhibition) ^2^	*h*HDAC8 IC_50_ (μM)
**37**	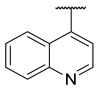	-CH-	3.57 ± 2.19	4.21	3.14 ± 1.01
**38**	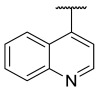	-N-	3.65 ± 2.39	4.42	1.74 ± 0.81
**39**	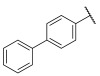	-N-	4.99 ± 1.82	5.35	4.29 ± 1.42

^1^ HeLa nuclear extract (rich in HDAC1 and HDAC2). ^2^ Percent inhibition of HDAC3 activity at 10 μM concentration.

**Table 9:** General structure and IC_50_ values of **48**, **49**, and **50** [156,157].

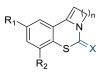									
Cpds	R_1_	R_2_	X	*n*	*h*HDAC1 IC_50_ (μM)	*h*HDAC2 IC_50_ (μM)	*h*HDAC3 IC_50_ (μM)	*h*HDAC6 IC_50_ (μM)	*h*HDAC8 IC_50_ (μM)
48	H-	H-	-N-	3	3.6 ± 0.8	32 ± 15	>50	6.7 ± 0.8	0.011 ± 0.001
49	H-	F-	-N-	3	35 ± 3	>50	>50	5.2 ± 1.1	0.017 ± 0.0001
50	Br-	H-	-S-	2	>50	>50	>50	>50	0.260

The predicted ZBGs are in blue.

## Data Availability

Not applicable.
